# Treg activation during allograft tolerance induction requires mitochondrion-induced TGF-**β**1 in type 1 conventional dendritic cells

**DOI:** 10.1172/JCI178960

**Published:** 2025-07-10

**Authors:** Samantha L. Schroth, Lei Zhang, Rebecca T.L. Jones, Kristofor Glinton, Nikita L. Mani, Hiroyasu Inui, Jesse T. Davidson, Samuel E. Weinberg, Navdeep S. Chandel, Maria-Luisa Alegre, Edward B. Thorp

**Affiliations:** 1 Northwestern University Feinberg School of Medicine, Chicago, Illinois, USA.; 2 State Key Laboratory of Natural Medicines, China Pharmaceutical University, Nanjing, China.; 3 Center for Human Immunbiology, and; 4 Department of Pathology, Feinberg School of Medicine, Chicago, Illinois, USA.; 5 Comprehensive Transplant Center, Feinberg School of Medicine, Chicago, Illinois, USA.; 6 Department of Medicine, Section of Rheumatology, University of Chicago, Chicago, Illinois, USA.

**Keywords:** Immunology, Transplantation, Dendritic cells

## Abstract

The role of conventional type 1 DCs (cDC1s) in tolerance induction to solid organ allografts is unknown and important for strategies that seek to prolong allograft viability. Using a murine model deficient in cDC1s, we report cDC1s are required for donor antigen and costimulation blockade (DST + CoB) tolerance induction and survival of cardiac allografts. cDC1 deficiency led to decreases in CD4^+^CD25^+^FoxP3^+^ T cells within allograft and spleen tissue of transplant recipients, and this was found to be antigen specific. Donor antigen stimulation induced TGF-β1 expression in both in vivo cDC1s and in vitro Flt3L-derived cDC1s. Genetic deletion of *TGF-β1* in cDC1s prevented induction of antigen-specific CD4^+^CD25^+^FoxP3^+^ T cells and was associated with cardiac allograft rejection. In parallel, single-cell RNA sequencing and metabolic analysis revealed upregulation of cDC1 mitochondrial metabolic signatures after in vivo exposure to DST + CoB. Genetic inactivation of cDC1 mitochondrial metabolism reduced expression of cDC1 TGF-β1, decreased antigen-specific Treg populations, and impaired allograft tolerance. Taken together, our findings implicate cDC1s in strategies to preserve solid organ allografts and also implicate mitochondrial metabolism of cDC1s as a molecular mechanism to enhance the generation of antigen-specific CD4^+^CD25^+^FoxP3^+^ T cells through TGF-β1.

## Abstract 2

This study newly implicates a dendritic cell subset and an underlying immunometabolic mechansiom during strategies for allograft tolerance

## Graphical abstract

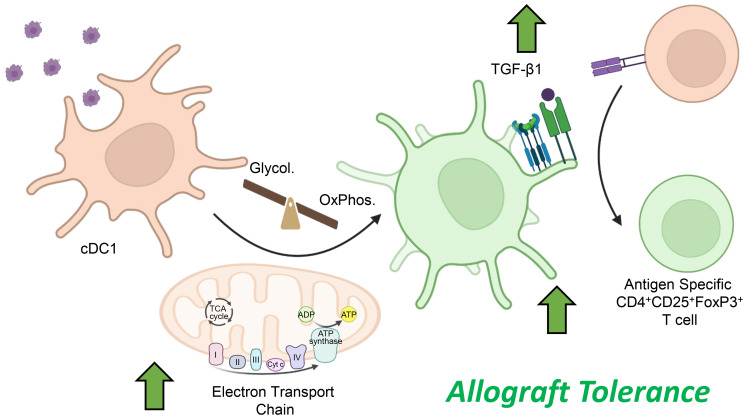

## Introduction

Heart transplantation remains the gold standard therapy for individuals with end-stage heart failure (1). Although acute patient survival after transplantation has improved significantly, the same cannot be said for chronic allograft survival (2, 3). Pathologies such as chronic allograft vasculopathy are perpetuated by a loss of immune tolerance and result in graft failure and significant morbidity and mortality for patients (4–6). Therefore, improved understanding of fundamental mechanisms of immune tolerance and regulation have the potential to inform strategies to promote survival of the allograft.

Conventional type 1 DCs (cDC1s) are powerful orchestrators of the adaptive (7–9) and innate (10, 11) immune response. As professional antigen-presenting cells, cDC1s are known for their canonical role in cross-presentation of antigen to CD8^+^ T cells (12), though more recent work has identified a critical role for cDC1s in the priming of CD4^+^ T cells (8). Within these interactions, cDC1s have been shown to promote both stimulatory (13, 14) and regulatory immune responses, including those relevant to self-tolerance (15–17). Additionally, uptake and clearance of apoptotic cells, also known as efferocytosis, is known to be antiinflammatory (18–20), though the relevance and role of efferocytic signaling in cDC1s, as well as the role of cDC1s in solid organ transplantation or allogenic tolerance, remains largely unknown. Here, we describe studies revealing a requirement of the cDC1 population for the prolongation of cardiac allograft survival mediated by the induction of a splenic antigen-specific CD4^+^CD25^+^FoxP3^+^ T cell population through cDC1-expressed TGF-β1 after exposure to allogenic donor antigen. Additionally, we identify a contributing role of mitochondrial metabolism for cDC1 tolerogenic reprogramming and TGF-β1 expression.

## Results

### cDC1s are critical for donor antigen plus CoB-mediated cardiac allograft survival.

**Figure 1:**
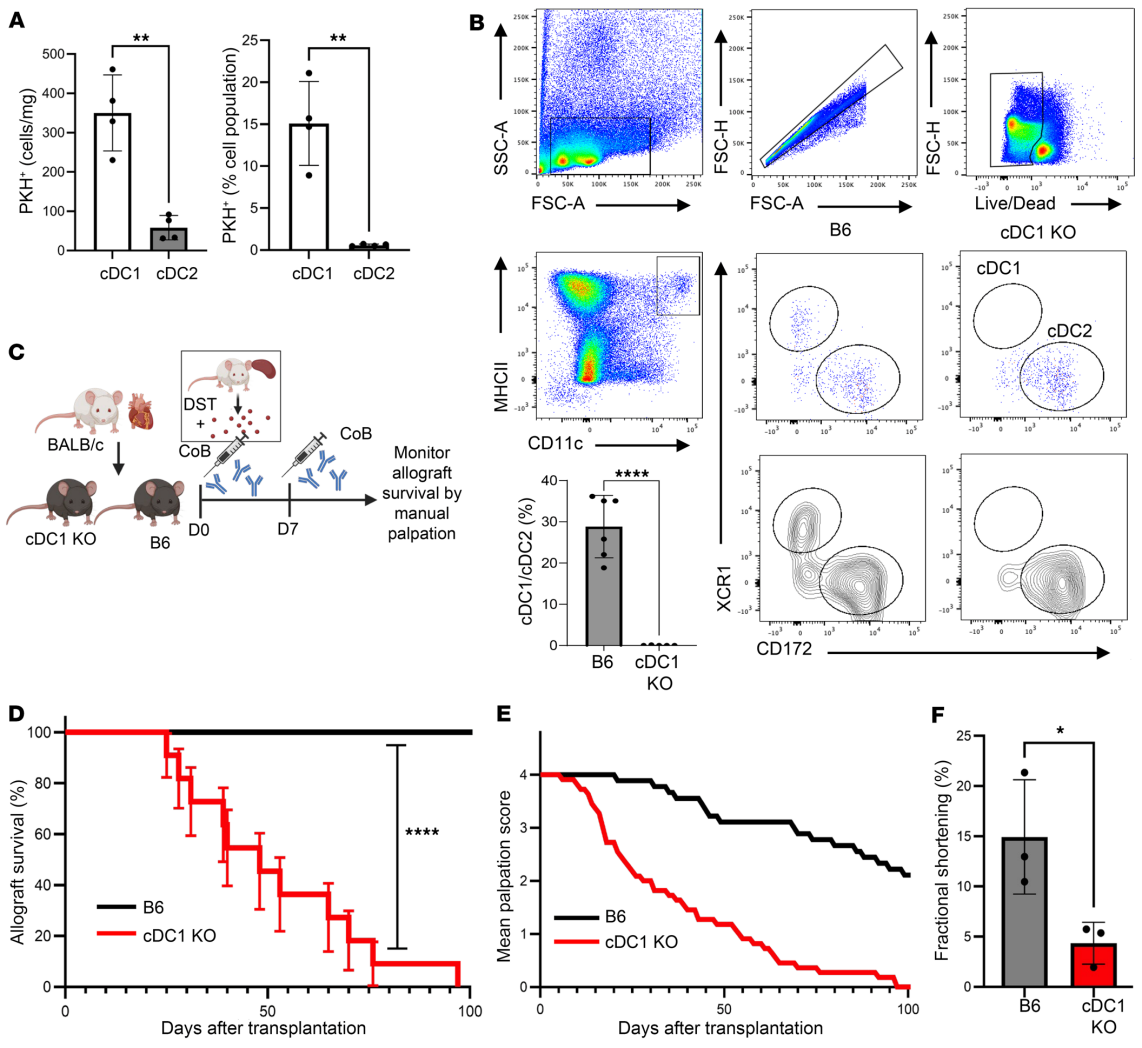
cDC1s are required for donor splenocyte transfusion and anti-CD40L costimulation blockade–mediated cardiac allograft survival. (**A**) Uptake of PKH membrane–labeled CD45.1^+^ donor splenocytes 18 hours after i.v. infusion by CD45.1^–^ B6 recipient splenic DCs measured by flow. *n* = 4 per group. **P < 0.01 by 2-tailed unpaired *t* test. (**B**) Flow cytometry gating strategy for cDC1 and cDC2 identification in spleens of B6 and cDC1-KO mice. Flow cytometry gating strategy to estimate the amount of splenic cDC1 and cDC2 cells in cDC1-KO and B6 mice as well as the ratio of cDC1/cDC2 cells. *n* = 6 per group. ****P < 0.0001 by 2-tailed unpaired *t* test. (**C**) Heterotopic heart transplantation experimental scheme. (**D**) Survival of BALB/c cardiac allografts in B6 and cDC1-KO mice as determined by manual palpation with rejection occurring at complete cessation of heartbeat and a palpation score of 0. *n* = 9–11 per group. ****P < 0.0001 by log-rank test. (**E**) Mean palpation score of BALB/c cardiac allografts in B6 and cDC1-KO mice. (**F**) Measurement of fractional shortening of cardiac allografts by echocardiography 42 days after transplantation. *n* = 3 per group. *P < 0.05 by 2-tailed unpaired *t* test.

Presentation of donor antigen by professional antigen-presenting cells to host T cells is a crucial component of the anti-donor response after transplantation (21), and CD11c^+^ DCs are known to internalize donor antigen, especially within the spleen as compared with other locations such as the lymph nodes or liver (22). The field of immunology has also come to appreciate the heterogeneity of DCs and more importantly, the subsequent identification of unique DC subsets, which differ in development, surface phenotype, and function (23, 24), and likewise play disparate roles within an immune response (17, 25). To identify localization of donor antigen to a specific population of antigen-presenting DCs, BALB/c splenocytes were labeled with a membrane fluorophore (PKH-67) and injected into C57BL/6J (B6) WT mice. The distribution of PKH-67^+^ cells 18 hours after injection was investigated by flow cytometry (Supplemental Figure 1; supplemental material available online with this article; https://doi.org/10.1172/JCI178960DS1), which revealed alloantigen was taken up by and localized predominantly within the XCR1^hi^ CD172^lo^ cDC1 subset (Figure 1A). Given these results, the known efficiency in which cDC1s present antigen to T cells, and recent findings indicating a role for cDC1s in self-tolerance (15–17), we hypothesized cDC1s are involved in the promotion of a tolerogenic response to a solid organ allograft.

**Figure 2:**
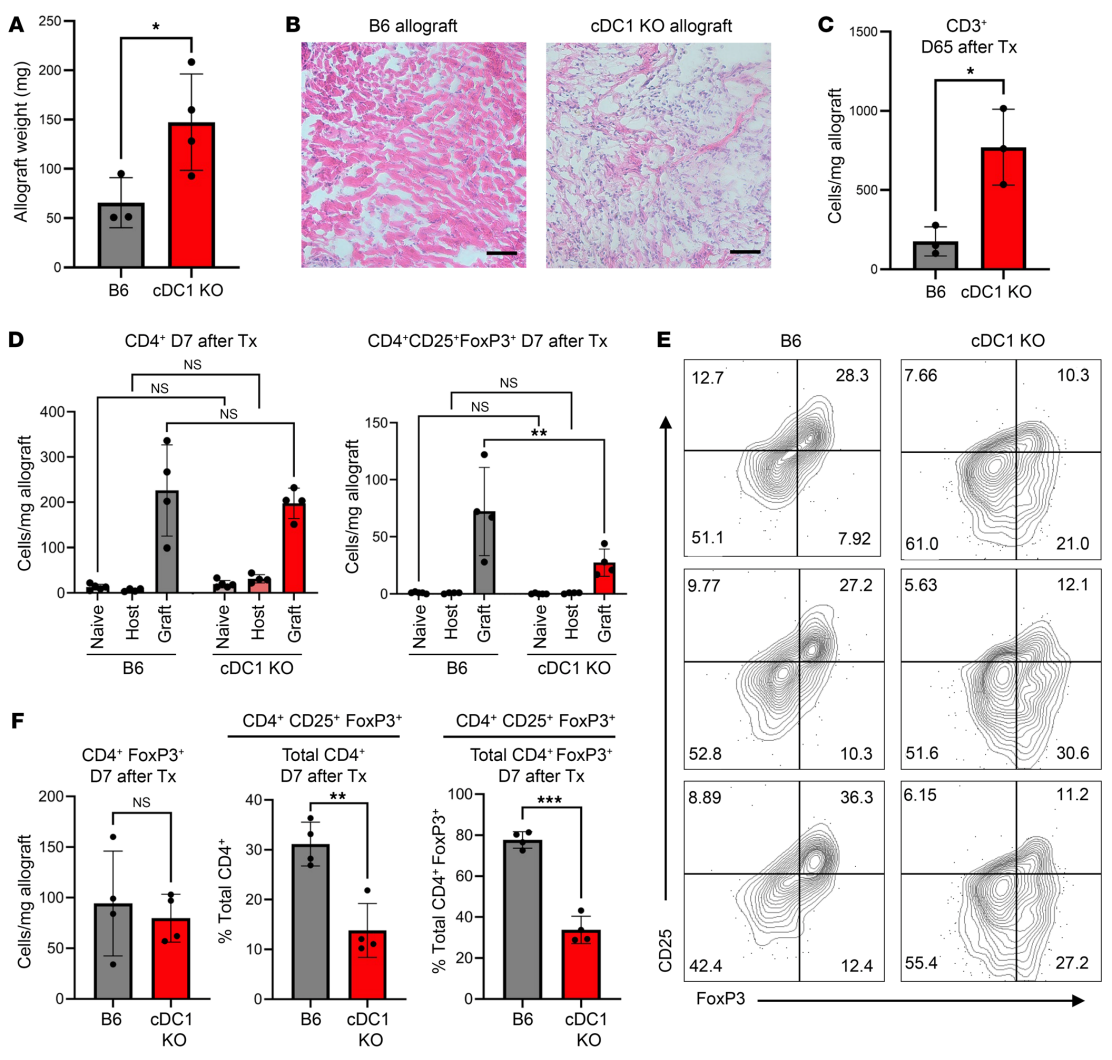
cDC1s are critical for CD25^+^FoxP3^+^ T cell induction in cardiac allograft in the setting of DST + CoB. (**A**) Weight of cardiac allografts 65 days after transplantation. *n* = 3–4 per group. *P < 0.05, by 2-tailed unpaired *t* test. (**B**) H&E staining of cardiac allografts 65 days after transplantation. Scale bar: 50 μm. (**C**) Quantification of CD3^+^ T cells in cardiac allografts 65 days after transplantation. *n* = 3 per group. *P < 0.05, by 2-tailed unpaired *t* test. (**D**) Quantification of CD4^+^ and CD4^+^CD25^+^FoxP3^+^ T cells in hearts of naive mice, recipient orthotopic (host) heart, and recipient heterotopic (allograft) heart 7 days after transplantation. *n* = 4 per group. **P < 0.01 by 1-way ANOVA followed by Tukey’s test. (**E**) Representative flow plots of CD25^+^FoxP3^+^ T cells in B6 and cDC1-KO cardiac allografts 7 days after transplantation. Cells were pre-gated as live single CD3^+^CD4^+^ cells. (**F**) Quantification of CD4^+^CD25^+^FoxP3^+^ T cells in recipient heterotopic (allograft) hearts 7 days after transplantation in B6 and cDC1-KO mice. Data shown as cells/mg allograft tissue and as the percentage of CD4^+^CD25^+^FoxP3^+^ T cells within CD4^+^ and CD4^+^FoxP3^+^ cell populations. *n* = 4 per group. **P < 0.01, ***P < 0.001 by 2-tailed unpaired *t* test.

Prior studies have detailed how transfusion of donor splenocyte antigen (DST) in combination with anti-CD40L CoB can induce long-term (>100 days) antigen-specific tolerance to a variety of solid organ allografts, including the cardiac allograft (26–29). We acquired an *Irf8* +32^–/–^ cDC1-KO mouse on a B6 background, which was genetically deficient in the cDC1 subset due to deletion of an enhancer 38 kb downstream of the IRF8 transcriptional start site required for cDC1 fate specification and development (30). We confirmed deletion of splenic cDC1s (Figure 1B) and subjected cDC1-KO and B6 mice to full-MHC mismatch heterotopic heart transplantation (31) utilizing BALB/c donors while employing a DST + CoB tolerization strategy whereby recipient mice received i.v. infusion of donor splenocytes and anti-CD40L (DST + CoB) on the day of transplant (day 0), followed by an additional dose (i.p.) of anti-CD40L on day 7 (Figure 1C, detailed in Methods). Recipient allograft function and survival were assessed and scored by manual palpation with allograft rejection identified as complete cessation of pulsation (32). Mice lacking cDC1s exhibited significantly earlier allograft rejection (Figure 1D) and a concomitant decrease in mean palpation score (Figure 1E) compared with immunocompetent B6 mice. We observed no sex differences in allograft survival within genotypes (Supplemental Figure 2). Echocardiography of allografts on day 42 after transplantation provided quantitative corroboration of clinical palpation scores as it revealed a concordant decrease in ventricular fractional shortening of cDC1-KO allografts (Figure 1F). Additionally, in cardiac allografts that were still functional at day 65 after transplantation prior to harvest, cDC1-KO allografts were found to be of increased weight (Figure 2A) and have a disorganized tissue structure with noticeable quantities of cellular infiltrates compared with B6 allografts (Figure 2B). Utilizing flow cytometry, we were able to further confirm and identify a significant increase in CD3^+^ T cells within cDC1-KO cardiac allografts at this time point (Figure 2C). It has been shown that treatment with anti-CD40L is able to not only prevent induction of alloantibodies but also dissolve established germinal centers (33). Given the growing body of work demonstrating the role B cells and antibody-mediated rejection can play in cardiac transplantation (34), we assessed peripheral blood for donor-specific antibodies and the quantity of CD19^+^ B cells in the spleen. The results showed no difference in CD19^+^ B cell numbers in cDC1-deficient transplant recipients (Supplemental Figure 3A) or in anti-BALB/c IgG levels across different dilution ratios compared with controls (Supplemental Figure 3B). Anti-BALB/c antibody levels in cDC1-KO mice were comparable to the B6 mice at day 65 after transplantation, though there was a trend toward decreased IgG in cDC1-KO recipients (Supplemental Figure 3C). These findings confirmed the allograft rejection was not antibody mediated.

### cDC1s are critical for activation of cardiac allograft CD4^+^FoxP3^+^ T cells.

As the causal importance of T cells in cardiac allograft rejection has been well established (35–37), and cDCs function in the necessary priming of alloreactive conventional T cells or Tregs (8, 12, 38, 39), we next sought to delineate the contribution of cDC1s in the early immune response to the cardiac allograft. We hypothesized it is the early priming by cDC1s of T cells toward a more tolerant versus activated phenotype that is critical for creating an immunological environment to ultimately allow for acceptance of the allograft. Although no difference in the number of CD4^+^ T cells was observed in the transplant recipients’ orthotopic or heterotopic allograft hearts 7 days after transplantation, we observed a significant decrease in CD4^+^CD25^+^FoxP3^+^ cells in the cardiac allograft of mice deficient in cDC1s (Figure 2, D and E). Closer analysis of the CD4^+^ T cell population revealed that, though both cDC1-KO and B6 WT mice had similar numbers of CD4^+^FoxP3^+^ cells (Figure 2, E and F), the proportion of these cells expressing the activation marker CD25, which is known to be important for the homeostasis and survival of Tregs (40), was significantly lower in cDC1-KO allografts. This remained true when comparing the proportion of CD4^+^CD25^+^FoxP3^+^ cells present within both the overall CD4^+^ T cell population and the smaller CD4^+^FoxP3^+^ population (Figure 2F). This phenotype was also observed within the spleens of cDC1-KO and B6 WT transplant recipient mice (Supplemental Figure 4).

### Antigen-specific regulatory CD4^+^ T cells are induced by cDC1s.

**Figure 3:**
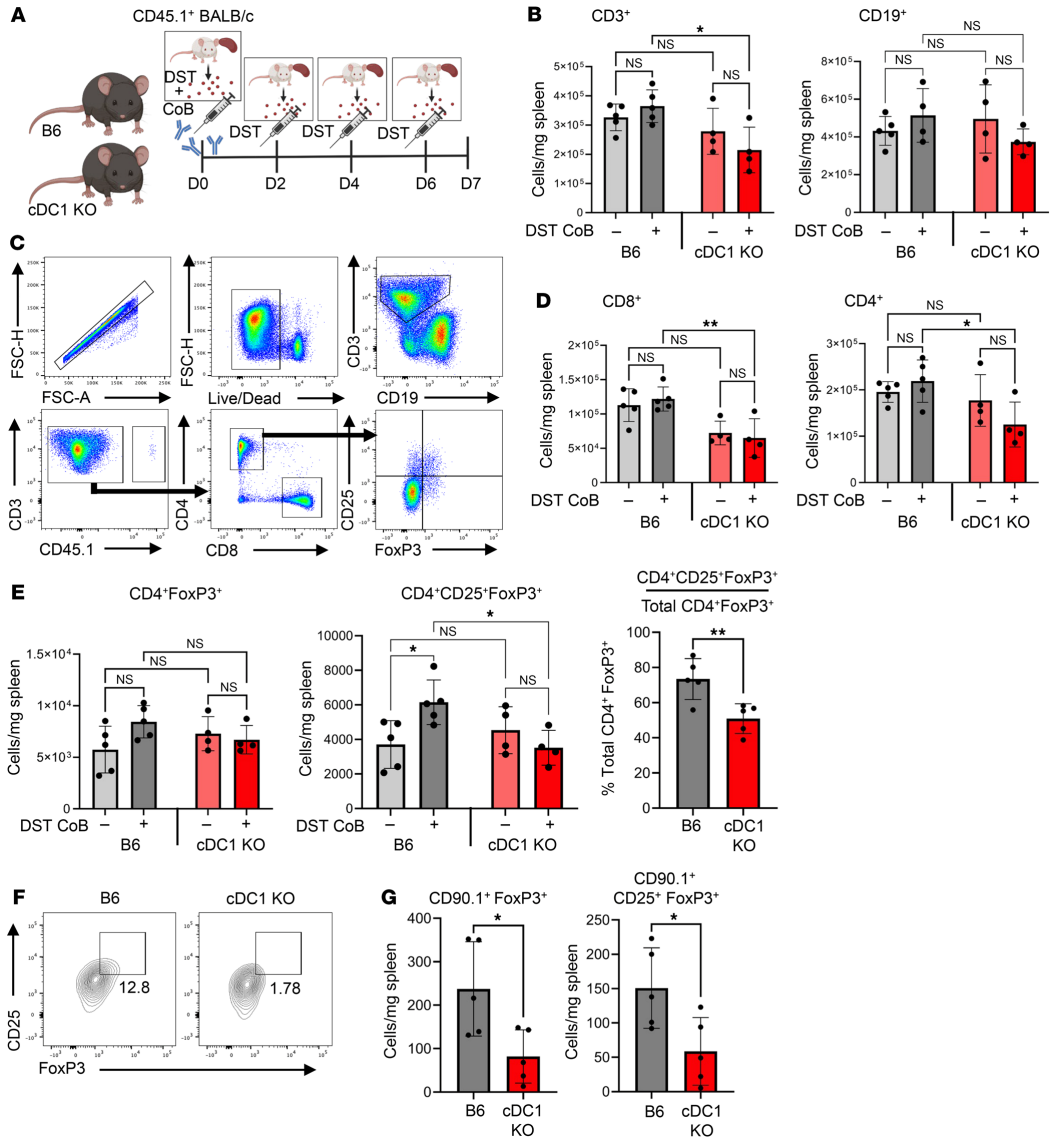
cDC1s are necessary for induction of splenic CD25^+^FoxP3^+^ T cells after DST + CoB. (**A**) Persistent antigen stimulation experimental scheme whereby B6 and cDC1-KO mice were treated with CD45.1^+^ BALB/c DST + CoB infusion on day 0 (i.v.), followed by CD45.1^+^ BALB/c DST injections (i.p.) on days 2, 4, and 6 before collection of spleens on day 7. (**B**) Quantification of CD45.1^–^ adaptive immune cell populations (CD3^+^ or CD19^+^) in spleens of naive and persistent antigen-treated B6 or cDC1-KO mice. *n* = 4–5 per group. *P < 0.05 by 1-way ANOVA with Tukey’s test followed by Tukey’s test. (**C**) Flow cytometry gating strategy for CD45.1^–^CD4^+^CD25^+^FoxP3^+^ T cell identification in spleens of B6 and cDC1-KO mice. (**D**) Quantification of CD45.1^–^CD8^+^ and CD45.1^–^CD4^+^ T cell populations in spleens of naive and persistent antigen-treated B6 or cDC1-KO mice. *n* = 4–5 per group. *P < 0.05, **P < 0.01 by 1-way ANOVA followed by Tukey’s test. (**E**) Quantification of CD4^+^FoxP3^+^ and CD4^+^CD25^+^FoxP3^+^ T cells in spleens of naive and persistent antigen-treated B6 or cDC1-KO mice. Cells were pre-gated as live single CD3^+^ CD4^+^ CD45.1^–^ cells. Data shown as cells/mg splenic tissue and as the percentage of CD4^+^FoxP3^+^ cell population. *n* = 4–5 per group. *P < 0.05, **P < 0.01 by 1-way ANOVA followed by Tukey’s test. (**F**) Congenic transfer of CD90.1^+^ OT-II CD4 T cells into B6 and cDC1-KO mice on day –1 followed by persistent membrane-bound ova + CoB and persistent ova antigen stimulation. Representative gating and flow plots of CD3^+^CD90.1^+^CD25^+^FoxP3^+^ OTII T cells specific to ova antigen in B6 versus cDC1-KO spleens. Cells were pre-gated as live single CD3^+^ CD90.1^+^ cells. (**G**) Quantification of CD90.1^+^FoxP3^+^ OTII and CD90.1^+^CD25^+^FoxP3^+^ OTII T cells in B6 or cDC1-KO mice after ova + CoB and persistent ova antigen stimulation. *n* = 5 per group. *P < 0.05 by 2-tailed unpaired *t* test.

Tregs, defined by their expression of CD25 and FoxP3, are of great interest within the transplant community as a result of their functional role in suppressing antigen-specific alloreactive T cells and promoting states of tolerance (41). Additionally, CD4^+^CD25^+^ regulatory cells have been shown to be induced by and required for DST + CoB–mediated allograft tolerance (42, 43). A variety of pathways by which peripheral CD4^+^ T cells become CD25^+^FoxP3^+^ Tregs have been described, though mechanistic gaps remain, particularly in the setting of alloantigen and solid organ transplantation (43–46). Given our interest in understanding the broader role cDC1s play in this induction of CD4^+^CD25^+^FoxP3^+^ Tregs that are responsive to alloantigen, we utilized a non–heart transplant model in which B6 WT and cDC1-KO mice received DST + CoB treatment on day 0 followed by persistent exposure to donor antigen through infusion of DST every other day until harvest at day 7 (47) (Figure 3A). Since T cell priming and antigen presentation by cDCs occurs predominately within lymph nodes and secondary lymphoid organs, we focused our studies within the spleen.

We observed no differences in the quantity of CD19^+^ B cells in B6 mice and cDC1-KO naive mice before or after DST + CoB with persistent antigen infusion, but we did observe a decrease in the number of splenic CD3^+^ T cells in cDC1-KO mice after treatment (Figure 3B). We sought to identify the subset of recipient T cells contributing to this observed difference (gating strategy Figure 3C) and observed a decrease in the number of CD8^+^ and CD4^+^ T cells in cDC1-KO mice compared with B6 mice after DST + CoB treatment (Figure 3D). Although the role of cDC1s in antigen presentation and stimulation of CD8^+^ T cells has been well characterized (7, 12), we were very intrigued to observe this decrease in CD4^+^ T cells given our prior results in which both cardiac allografts and spleens of cDC1-KO mice had fewer CD4^+^CD25^+^FoxP3^+^ T cells 7 days after transplantation. Indeed, we observed the same results in which no difference in CD4^+^FoxP3^+^ T cell numbers was observed after DST + CoB and persistent antigen infusion; however, a significant decrease in the absolute quantity of splenic CD4^+^CD25^+^FoxP3^+^ T cells was seen in treated cDC1-KO mice (Figure 3E). We also characterized cells of the innate immune system in this setting and observed no differences in the quantity of cells in naive mice or mice treated with DST + CoB and persistent antigen infusion (Supplemental Figure 5).

Given that it was the specific subset of CD25^+^FoxP3^+^ T cells affected by cDC1 deficiency after both transplantation and alloantigen exposure, and that CD25 is a marker of T cell activation and proliferation in both regulatory and effector T cells (48), we hypothesized cDC1s may be functioning in an antigen-specific manner to promote and activate this regulatory response to alloantigen. Therefore, we transferred congenic CD90.1^+^ OT-II splenic CD4^+^ T cells, which recognize ovalbumin (ova) presented by I-A^b^ MHC class II into B6 and cDC1-KO mice on day –1, a strategy proven to efficiently promote T cell activation. We subsequently treated these mice with our DST + CoB and persistent antigen infusion protocol using splenocytes from mice constitutively expressing membrane-bound ova for each DST infusion. Indeed, when assessing CD90.1^+^ ova antigen-specific T cells, we observed fewer FoxP3^+^ and CD25^+^FoxP3^+^ antigen-specific cells in cDC1-KO mice (Figure 3, F and G), suggesting that cDC1s are important in the conversion of OTII conventional T cells into Tregs or in the expansion of OTII Tregs after CoB.

### Expression of TGF-β1 is increased in cDC1s after exposure to alloantigen in the setting of costimulation blockade.

**Figure 4:**
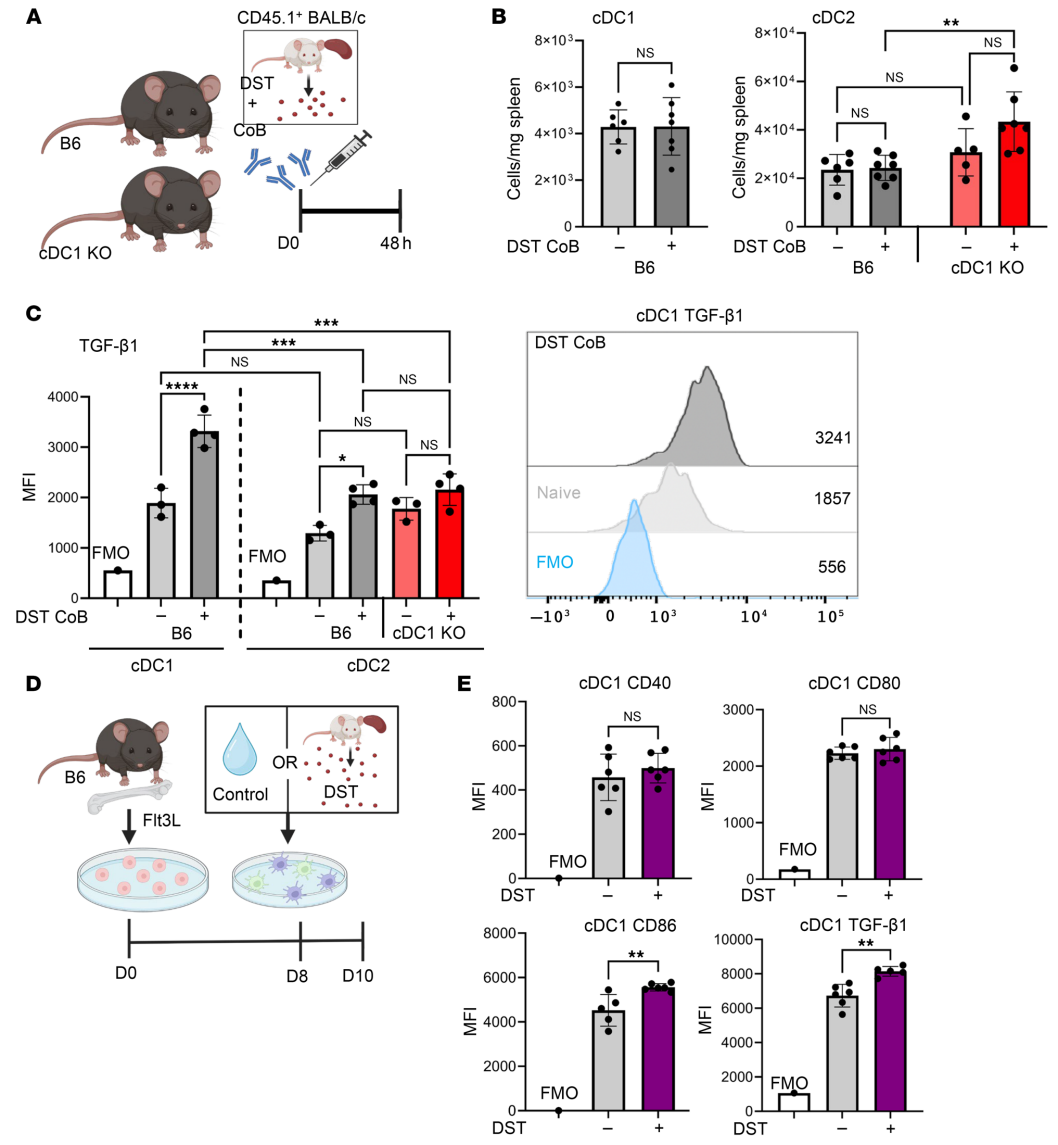
After exposure to allogenic cells, cDC1s increase expression of TGF-β1. (**A**) Acute DST + CoB experimental scheme whereby B6 and cDC1-KO mice were treated with CD45.1^+^ BALB/c DST + CoB infusion D0 (i.v.) before collection of spleens after 48 hours. (**B**) Quantification of cDC1 (Xcr1^+^CD172^–^) and cDC2 (Xcr1^–^CD172^+^) cell populations in spleens of naive and DST + CoB–treated B6 or cDC1-KO mice. Cells were pre-gated as live single CD11c^+^MHCII^hi^ cells. *n* = 5–7 per group. **P < 0.01 by 1-way ANOVA followed by Tukey’s test. (**C**) Expression of TGF-β1 in cDC1 and cDC2 splenic cells in naive and acute DST + CoB–treated mice. *n* = 3–4 per group. *P < 0.05, ***P < 0.001, ****P < 0.0001 by 1-way ANOVA followed by Tukey’s test. (**D**) In vitro allogenic stimulation experimental scheme whereby Flt3L BM-derived DCs were cultured for 8 days and exposed to CD45.1^+^ BALB/c DST or saline control for 48 hours. (**E**) Expression of canonical markers of DC activation (CD40, CD80, CD86) and TGF-β1 of cultured cDC1s following allogenic cell exposure. *n* = 5–6 per group. **P < 0.01 by 2-tailed unpaired *t* test. FMO, fluorescence minus one.

Because our results continued to emphasize the importance of cDC1s in the induction of antigen-specific CD4^+^CD25^+^FoxP3^+^ T cells, we sought to delineate the means by which cDC1s promote this response after exposure to alloantigen. Thus, we infused DST + CoB into B6 and cDC1-KO mice and assessed the phenotype of treated versus naive splenic DCs (Figure 4A). We saw no differences in cDC1 or cDC2 cell numbers after DST + CoB infusion when compared with control injection in WT mice, but did observe a slight increase in the number of cDC2s after DST + CoB in cDC1-KO mice (Figure 4B). As we began to evaluate candidate tolerogenic or regulatory ligands on cDC subsets, we identified a notable increase in cDC1 expression of membrane-bound TGF-β1 after DST + CoB treatment (Figure 4C). Interestingly, this intensity of TGF-β1 expression was higher in cDC1s when compared with cDC2s (Figure 4C), which may indicate a distinct function of this DC subset.

Although these results highlight an important in vivo response, it is important to acknowledge that these DCs were likely subjected to a variety of additional environmental signals aside from simple exposure to alloantigen, including crosstalk with other immune cells. Therefore, we wanted to see whether this induction of TGF-β1 expression was cell-intrinsic and could be observed when DCs were isolated and exposed to alloantigen in a controlled cellular environment. It is now well accepted that the receptor tyrosine kinase Flt3 is required for DC development (49); thus, in vitro culture of BM-derived DCs (BMDCs) must utilize Flt3 ligand (Flt3L) to generate DC subsets with biological relevance that mimic an in vivo response (50). As such, we used the Flt3L culture system to generate a high quantity of biologically relevant DCs (Supplemental Figure 6A). After differentiation, BMDCs were exposed to CD45.1^+^ BALB/c DST or saline and harvested 48 hours later (Figure 4D). Within the cDC1 population, we observed no changes in the expression of canonical markers of DC activation, including CD40 and CD80, but did identify an increased expression of cDC1 CD86 and TGF-β1 (Figure 4E and Supplemental Figure 6B).

### Membrane-bound cDC1 TGF-β1 is necessary for antigen-specific induction of regulatory CD4^+^ T cells.

TGF-β1 is known to induce *Foxp3* gene expression, to mediate the conversion of CD4^+^CD25^–^ conventional T cells into a CD4^+^CD25^+^ Treg population in vitro, and to be critical for generation of the Treg population and T cell tolerance in vivo (48, 51, 52). Additionally, membrane-bound TGF-β1 on CD4^+^ T cells and human DCs has been reported to have immunoregulatory functions in contact-dependent suppression of an effector cell population (53–55). Thanks to new genetic tools that allow for specific deletion of genes in the cDC1 cell population (Xcr1^Cre/+^) (8), we crossed Xcr1^Cre/+^ mice with mice containing loxP flanked sites on exon 3 of the *Tgfb1* gene (TGF-β1^fl/fl^) to delete membrane-bound TGF-β1. Xcr1^cre/+^ TGF-β1^fl/fl^ mice retained normal numbers of cDC1 and cDC2 cells in the spleen (Supplemental Figure 7, A and B). Additionally, the levels of CD40, CD80, and CD86 were similar between Xcr1^cre/+^ TGF-β1^fl/fl^ and TGF-β1^fl/fl^ mice (Supplemental Figure 7C), suggesting that TGF-β1 deficiency did not affect cDC1 development.

**Figure 5:**
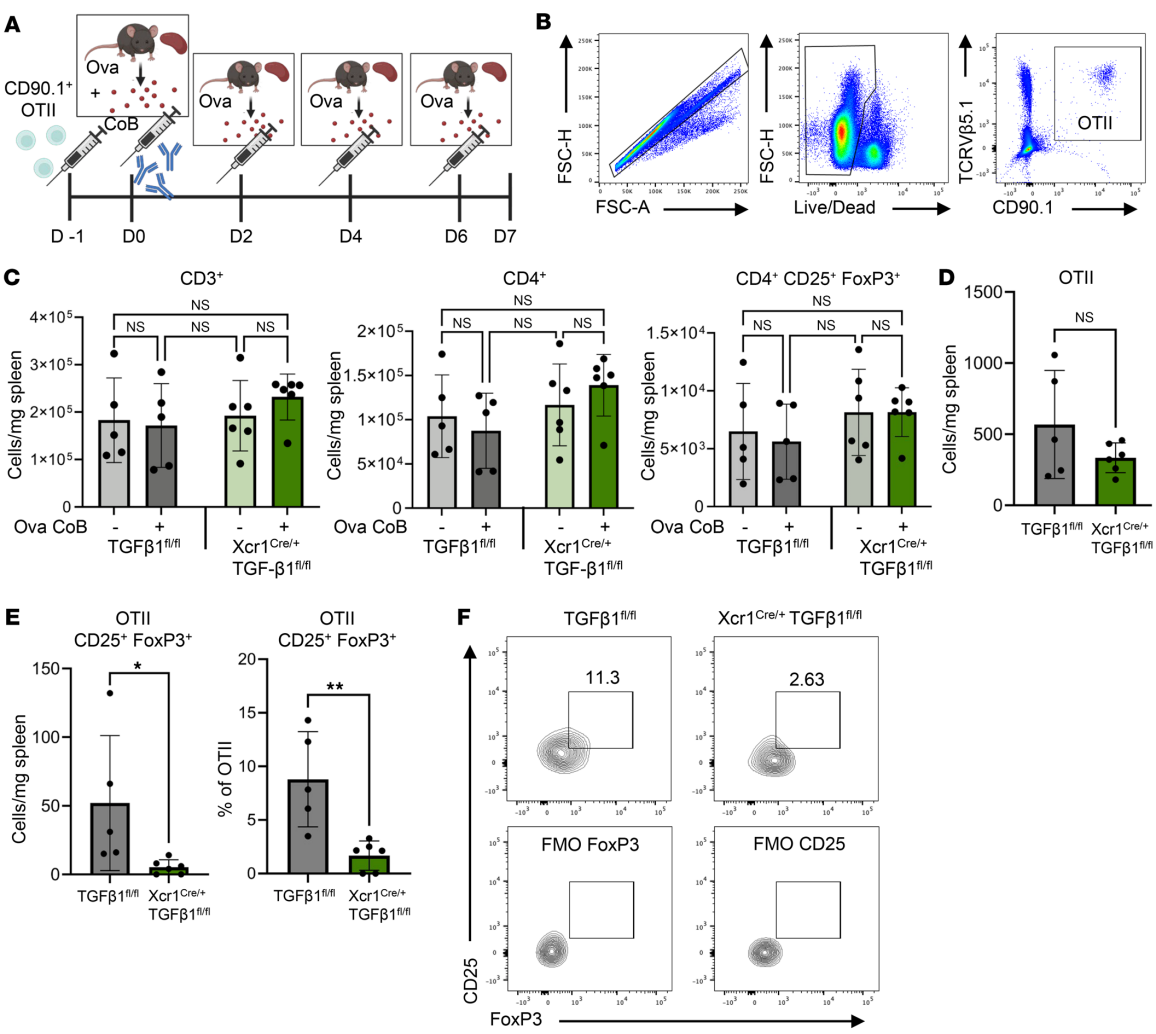
cDC1-expressed TGF-β1 is necessary for induction of antigen-specific CD25^+^FoxP3^+^ T cells. (**A**) Antigen-specific persistent antigen simulation experimental scheme whereby TGF-β1^fl/fl^ and Xcr1^Cre/+^TGF-β1^fl/fl^ mice were injected (i.v.) with CD90.1^+^ OTII T cells on day –1 and treated with ova DST + CoB infusion (i.v.) on day 0, followed by ova DST injections (i.p.) on days 2, 4, and 6 before collection of spleens on day 7. (**B**) Flow cytometry gating strategy for identification of splenic antigen-specific congenic CD90.1^+^ OTII cells from the spleens of ova DST + CoB–treated mice. (**C**) Quantification of endogenous splenic CD3^+^, CD4^+^, and CD4^+^CD25^+^FoxP3^+^ T cells. *n* = 5–6 per group. Determined no significance (ns) by 1-way ANOVA followed by Tukey’s test. (**D**) Quantification of splenic CD90.1^+^ OTII T cells 7 days after ova DST + CoB and persistent ova antigen treatment. *n* = 5–6 per group. Determined no significance (ns) by 2-tailed unpaired *t* test. (**E**) Quantification of splenic CD90.1^+^ OTII CD25^+^FoxP3^+^ T cells. Data shown as cells/mg splenic tissue and as the percentage of OTII cell population. *n* = 5–6 per group. *P < 0.05, **P < 0.01 by 2-tailed unpaired *t* test. (**F**) Representative flow plots of OTII CD25^+^FoxP3^+^ T cells in spleens of persistent antigen-treated TGF-β1^fl/fl^ and Xcr1^Cre/+^TGF-β1^fl/fl^ mice. Cells were pre-gated as live single CD90.1^+^ TCRVβ5.1^+^ cells. FMO, fluorescence minus one.

We subsequently performed an adoptive transfer of antigen-specific CD4^+^ T cells with persistent antigen exposure protocol: CD90.1^+^ OT-II splenic CD4^+^ T cells were infused into TGF-β1^fl/fl^ and Xcr1^Cre/+^ TGF-β1^fl/fl^ mice on day –1 followed by treatment with ova mouse DST + CoB and persistent ova antigen infusions for 1 week (Figure 5A). We then assessed the antigen-specific response of the transferred CD90.1^+^ OTII cells (Figure 5B). Notably, no difference was observed between naive or treated TGF-β1^fl/fl^ and Xcr1^Cre/+^TGF-β1^fl/fl^ mice in the overall splenic CD3^+^, CD4^+^, and CD4^+^CD25^+^FoxP3^+^ T cell populations (Figure 5C), largely because this is an isogenic infusion. Although a similar number of CD90.1^+^ OTII cells were recovered from spleens of TGF-β1^fl/fl^ and Xcr1^Cre/+^TGF-β1^fl/fl^ treated mice (Figure 5D), we did, indeed, observe a significant decrease of ova antigen-specific OTII CD25^+^FoxP3^+^ T cells in terms of both the absolute cell number and as a percentage of the OTII cell population (Figure 5, E and F). Moreover, Xcr1^Cre/+^ TGF-β1^fl/fl^ mice indeed rejected their allograft earlier than TGF-β1^fl/fl^ mice (Supplemental Figure 7, D and E). We further confirmed the importance of TGF-β in in vitro induction of naive CD4 T cells into CD4^+^CD25^+^FoxP3^+^ Treg cells by Flt3L BMDC coculture. BMDCs were either stimulated with saline or UV-irradiated BALB/c DST for 48 hours and then cocultured with anti-CD3/CD28 plate-bound naive CD4 T cells supplemented with Il-2 in the presence of saline or anti–TGF-β neutralizing antibody (Supplemental Figure 8A). After a 5-day coculture, DST-stimulated BMDCs had increased induction of CD4^+^CD25^+^FoxP3^+^ Treg cells compared with the saline BMDC-treated control; however, this induction was ablated in anti-TGF-β–treated cocultured cells (Supplemental Figure 8, B and C). Furthermore, anti-TGF-β–treated CD4^+^CD25^+^FoxP3^+^ T cells had reduced expression of PD-1 (CD279) compared with DST-stimulated BMDC coculture alone (Supplemental Figure 8D). Interestingly, PD-1 expression has been shown to be associated with antigen-specific TCR activation of CD25^+^ Tregs in a manner similar to CTLA-4 expression and to be associated with CD44 expression (56). TGF-β1 has also been observed to induce PD-1 expression in T cells via *Smad3* (57). These results indicate a requirement of cDC1 membrane-bound TGF-β1 as a mechanism for induction and function of antigen-specific CD4^+^CD25^+^FoxP3^+^ T cells and allograft tolerance.

### Mitochondrial metabolism is increased in cDC1s after exposure to alloantigen in the setting of costimulation blockade.

**Figure 6:**
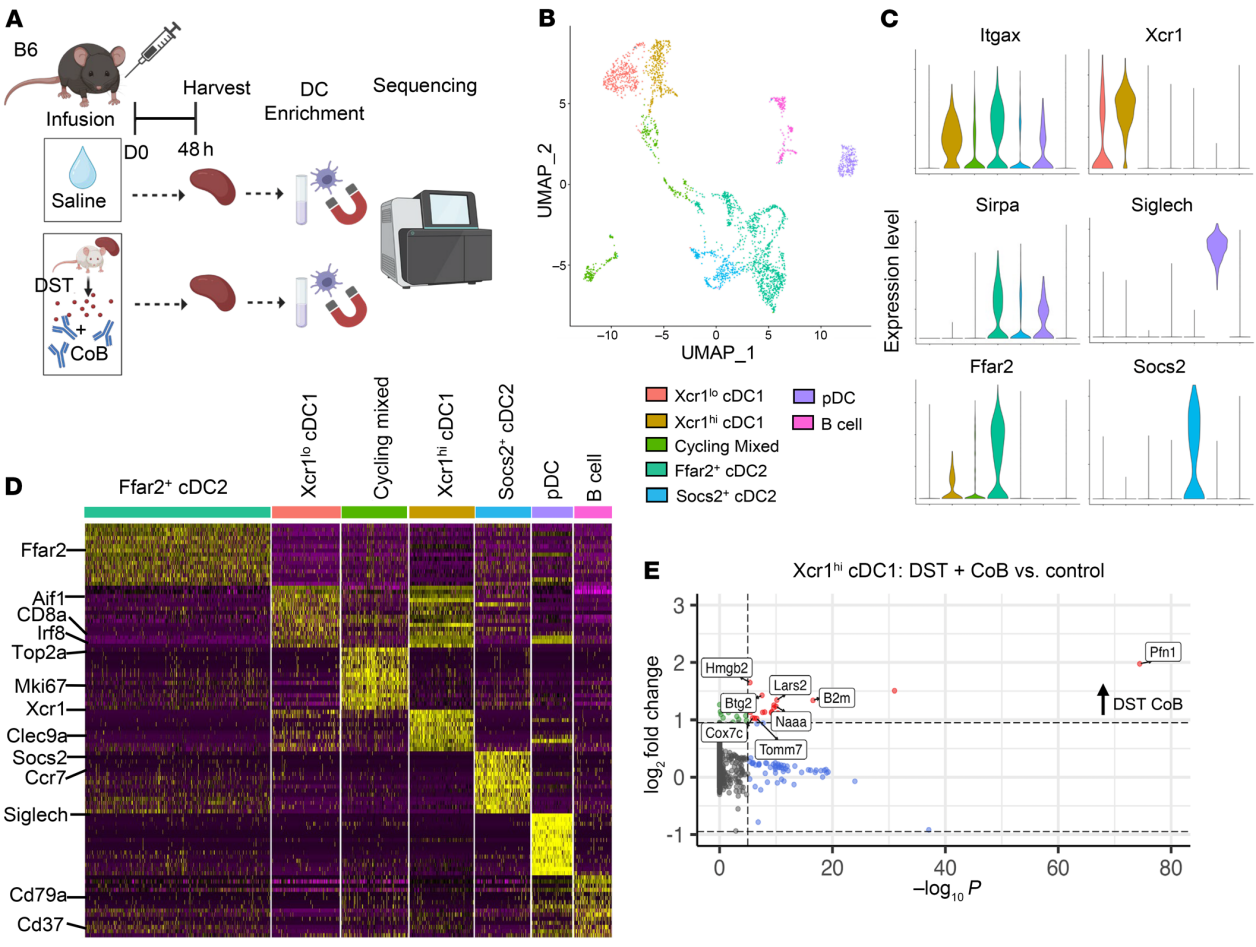
Conventional DC subsets display unique transcriptional signatures that are influenced by DST + CoB treatment. (**A**) Single-cell sequencing experimental scheme whereby B6 mice were treated with CD45.1^+^ BALB/c DST + CoB infusion (i.v.) on day 0 or saline control before collection of spleens after 48 hours, enrichment for DCs, and sequencing. (**B**) UMAP projections and identification of DC clusters, color-coded by cluster. (**C**) Violin plots of DC signature genes specifically expressed in their respective DC cluster. (**D**) Heatmap showing relative expression of marker genes across DC clusters. (**E**) Volcano plot of differentially expressed genes (DEGs) within Xcr1^hi^ cDC1 cluster following DST + CoB treatment compared with control.

Much work has been done to understand the downstream signaling by TGF-β1 in a variety of immune cell types and biological contexts, but we continue to seek to understand the means by which TGF-β1 secretion or expression occurs. Though some work has been done to understand how DCs are influenced by and respond to allogeneic antigen in vivo to promote an activating or regulatory cell response (22, 26, 58), questions remain in identifying the cell-intrinsic transcriptional reprogramming that occurs in cDC1s within this unique setting. Therefore, we performed single-cell RNA sequencing of DC-enriched splenocytes of WT B6 mice 48 hours after receiving BALB/c DST + CoB or saline infusion utilizing the 10x Genomics platform (Figure 6A). After quality control, filtering, normalization, and integration of the data, we employed uniform manifold approximation and projection (UMAP) dimensionality reduction analysis combined with unbiased cell type recognition utilizing the ImmGen open-source reference database (59) and *SingleR* algorithm to confirm cell cluster identity (Supplemental Figure 9). We then reclustered a subset of DC-identified clusters for further downstream analysis. A total of 2,432 individual cells were analyzed between the control and DST + CoB infusion conditions, which clustered into 7 distinct clusters expressing canonical markers for their respective cellular identity including *Xcr1*, *Sirpa*, *Siglech*, Ebf1, and Pax5 (Figure 6, B and C, and Supplemental Figure 10).

**Figure 7:**
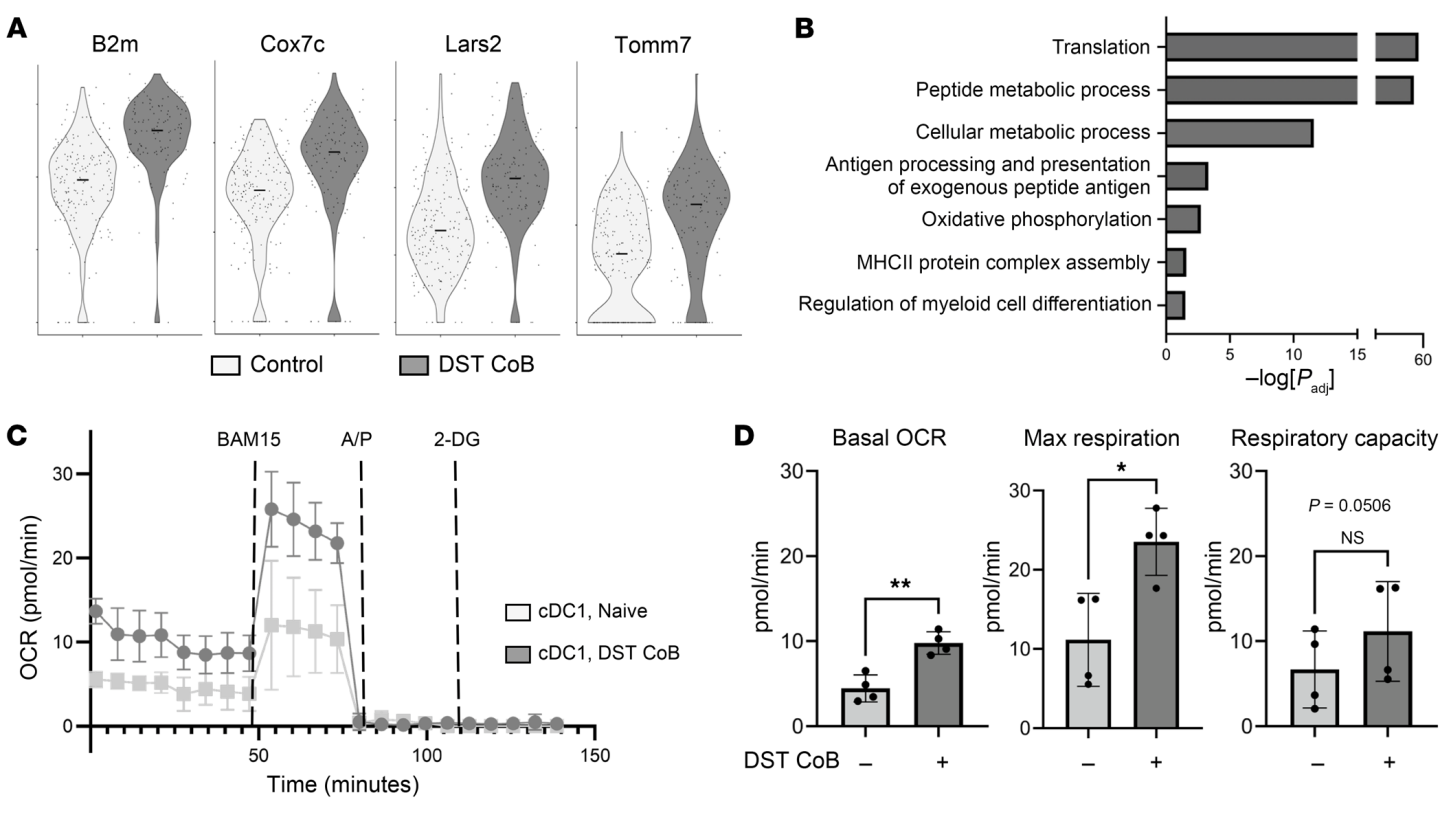
Mitochondrial respiration is increased in cDC1s after exposure to DST + CoB. (**A**) Violin plots of antigen presentation (B2m) and metabolism-related genes (Cox7c, Lars2, Tomm7) in Xcr1^hi^ cDC1s in control and DST + CoB infusion conditions by single-cell sequencing. (**B**) Gene ontology analysis of differentially expressed genes in Xcr1^hi^ cDC1s after DST + CoB compared with controls by single-cell sequencing. (**C**) Mitochondrial respiration of sorted splenic cDC1s 48 hours after DST + CoB treatment. *n* = 4 per group. A/P, antimycin A and piericidin A; 2-DG, 2-deoxy-D-glucose; OCR, oxygen consumption rate. (**D**) Quantification of basal OCR, max respiration, and respiratory capacity of naive and DST + CoB–treated cDC1s. *n* = 4 per group. *P < 0.05, **P < 0.01 by 2-tailed unpaired *t* test.

Two clusters of cDC1s were identified, which differed in their relative expression of *Itgax* and *Xcr1*. Given that expression of *Xcr1* is both selective and specific to cDC1s, we hypothesized that cells with lower levels of *Xcr1* expression were less mature and less likely to be participating in the immunoregulatory responses we observed. As such, we performed differential gene expression analysis of the *Xcr1^hi^* cDC1 cluster in control and DST + CoB infusion conditions (Figure 6D). We saw a significant increase in genes such as *Pfn1* thought to be related to cell migration and motility, as well as those related to cell proliferation, like *Btg2* (Figure 6E). We also noticed a number of genes related to antigen presentation (*B2m*) and metabolism (*Lars2*, *Cox7c*, *Naaa*), which were noticeably increased in *Xcr1^hi^* cDC1s exposed to DST + CoB in vivo (Figure 6E and Figure 7A). Therefore, we performed pathway analysis of the differentially expressed genes found within the *Xcr1^hi^* cDC1 cluster after DST + CoB by utilizing g:Profiler and Gene Ontology biological processes (60). We observed numerous processes we would expect for DCs exposed to antigen, including translation, antigen processing and presentation, and regulation of myeloid cell differentiation, in addition to numerous metabolic pathways (Figure 7B).

The canonical role of mitochondria and cellular metabolism for the generation of energy is well appreciated; however, in the more recent past, mitochondria are now lauded for their role as critical signaling organelles, especially as it relates to control of the innate and adaptive immune response (61). Studies have begun to investigate the role of metabolism in DCs during different biological states, but few experiments have been performed utilizing DCs isolated from in vivo tissue given low cell numbers and challenges with isolation and sorting protocols that may falsely activate or alternatively damage these fragile cells (62). Therefore, we developed a protocol utilizing a low-pressure, cartridge-based microfluidic cell sorter to isolate a highly pure population (>90%) of splenic cDC1s (Supplemental Figure 11). Using this protocol, we isolated cDC1s from spleens of B6 mice 48 hours after infusion of DST + CoB or saline and subjected them to metabolic analysis. As predicted by transcriptional sequencing, cDC1s exposed to DST + CoB displayed a higher oxygen consumption rate (OCR) compared with controls (Figure 7, C and D), which is a measure of mitochondrial oxidative phosphorylation. Likewise, cDC1s exposed to DST + CoB showed higher basal OCR and max respiration, as well as trends of increased respiratory capacity (Figure 7D). Importantly, no difference in mitochondrial mass of cDC1s was observed after DST + CoB treatment (Supplemental Figure 12), implying the observed results are indeed due to an increase in cDC1 mitochondrial activity.

### cDC1s require mitochondrial metabolism for TGF-β1–mediated antigen-specific induction of Tregs.

**Figure 8:**
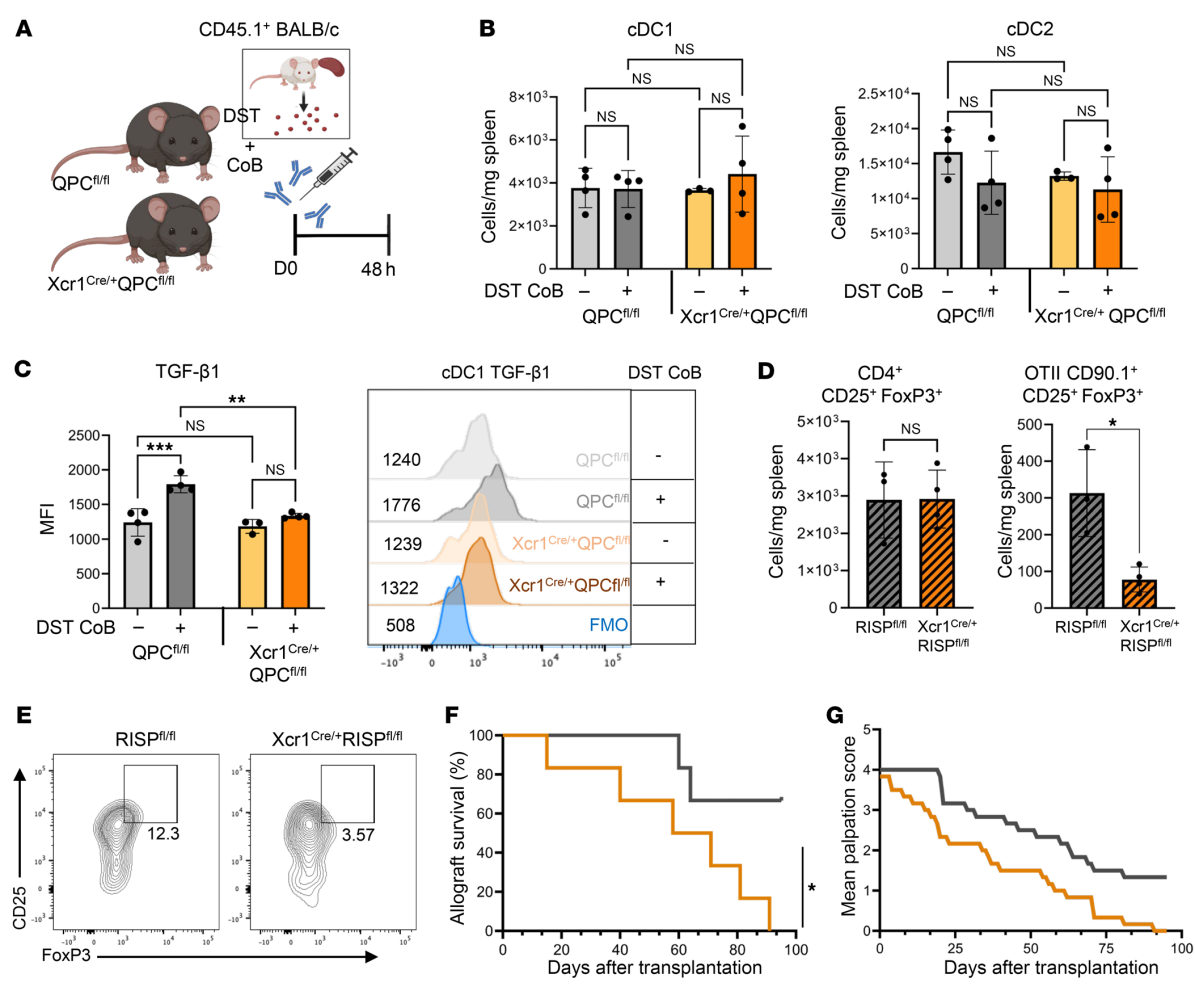
Mitochondrial respiratory transport chain complex III is required for induction of cDC1 TGF-β1 expression and antigen-specific CD25^+^FoxP3^+^ T cells after DST + CoB. (**A**) Acute DST + CoB experimental scheme whereby QPC^fl/fl^ and Xcr1^Cre/+^ QPC^fl/fl^ mice were treated with CD45.1^+^ BALB/c DST + CoB infusion (i.v.) on day 0 before collection of spleens after 48 hours. (**B**) Quantification of cDC1 (Xcr1^+^CD172^–^) and cDC2 (Xcr1^–^CD172^+^) cell populations in spleens of naive and DST + CoB–treated QPC^fl/fl^ and XCR1^Cre/+^QPC^fl/fl^ mice. Cells were pre-gated as live single CD45.1^–^CD11c^+^MHCII^hi^ cells. *n* = 3–4 per group. No significance (ns) by 1-way ANOVA followed by Tukey’s test. (**C**) Expression of TGF-β1 in cDC1 splenic cells in naive and acute DST + CoB–treated mice. *n* = 3–4 per group. **P < 0.01, ***P < 0.001 by 1-way ANOVA followed by Tukey’s test. Representative histogram of cDC1 TGF-β1 MFI. (**D**) Quantification of splenic CD25^+^FoxP3^+^ and CD90.1^+^ OTII CD25^+^FoxP3^+^ T cells in persistent antigen-treated RISP^fl/fl^ and Xcr1^Cre/+^RISP^fl/fl^ mice. *n* = 3–4 per group. *P < 0.05. Cells were pre-gated as live single CD25^+^FoxP3^+^CD4^+^ cells. (**E**) Representative flow plots of OTII CD25^+^FoxP3^+^ T cells in spleens of persistent antigen-treated RISP^fl/fl^ and Xcr1^Cre/+^RISP^fl/fl^ mice. Cells were pre-gated as live single CD90.1^+^ TCRVβ5.1^+^ cells. (**F**) Survival of BALB/c cardiac allografts in QPC^fl/fl^ and Xcr1^Cre/+^ QPC^fl/fl^ mice as determined by manual palpation with rejection occurring at complete cessation of heartbeat and a palpation score of 0. *n* = 6 per group. *P < 0.05 by log-rank test. (**G**) Mean palpation score of BALB/c cardiac allografts in QPC^fl/fl^ and Xcr1^Cre/+^ QPC^fl/fl^ mice.

In order to test the influence of mitochondrial metabolism of cDC1s in the setting of alloantigen in vivo, we crossed cDC1-specific Xcr1^Cre/+^ mice to mice with a loxP-flanked *Uqcsrf1* gene that encodes for Rieske iron-sulfur protein (RISP^fl/fl^) (63) and mice with a loxP-flanked *Uqcrq* gene that encodes for QPC protein (QPC^fl/fl^) (64), both subunits of mitochondrial complex III. After treatment of Xcr1^Cre/+^QPC^fl/fl^ and QPC^fl/fl^ control mice with DST + CoB or saline infusions (Figure 8A), we observed no differences in cDC1 or cDC2 cell number (Figure 8B) between control mice and those with cDC1s deficient in mitochondrial metabolism at both naive and posttreatment states. Additionally, we observed that QPC deficiency was associated with a slightly decreased expression of CD40 and IL10 but had no effects on CD80 and MHCII expression (Supplemental Figure 13). However, cDC1s deficient in mitochondrial metabolism did not increase their expression of TGF-β1 after DST + CoB treatment, as was observed in QPC^fl/fl^ controls (Figure 8C). We thus sought to determine whether this cDC1 deficiency of mitochondrial metabolism and subsequent decrease in TGF-β1 expression would affect the antigen-specific induction of CD4^+^CD25^+^FoxP3^+^ T cells. Therefore, we subjected Xcr1^Cre/+^RISP^fl/fl^ and RISP^fl/fl^ mice to our previously described protocol of CD90.1^+^ OTII cell transfer and persistent antigen infusions (Figure 5A). There was no influence of mitochondrion-deficient cDC1s on the overall population of CD4^+^CD25^+^FoxP3^+^ T cells after persistent antigen exposure; however, we did observe a significant decrease in antigen-specific CD90.1^+^ OTII CD25^+^FoxP3^+^ T cells (Figure 8, D and E). In addition, genetic inactivation of cDC1 mitochondrial metabolism, via Xcr1^Cre/+^QPC^fl/fl^, indeed impaired allograft tolerance after heterotopic transplantation compared with control QPC^fl/fl^ (Figure 8, F and G). These findings indicate the necessity of mitochondrial metabolism in cDC1s for increased expression of TGF-β1 for the antigen-specific induction of CD4^+^CD25^+^FoxP3^+^ T cells and allograft tolerance.

## Discussion

Taken together, our findings reveal a critical role for cDC1s in the promotion of an immunoregulatory environment through the induction of antigen-specific CD4^+^CD25^+^FoxP3^+^ T cells known to be critical in settings of self- and allotolerance (42, 45). We also report that cDC1s are the predominant professional antigen-presenting cell population to phagocytose and uptake alloantigen, which subsequently prompts cell-intrinsic transcriptional reprogramming, leading to mitochondrial metabolic signaling that promotes TGF-β1 expression necessary for induction of antigen-specific CD4^+^CD25^+^FoxP3^+^ T cells. The physiological process of efferocytosis and clearance of apoptotic cells by phagocytosing cells is recognized to be antiinflammatory in nature (18, 19), and these results indicate this paradigm is conserved within cDC1s. Additionally, our results have clear implications in the setting of alloantigen and transplantation that were explored, and they are likely relevant to other pathologies such as autoimmunity and cancer. Thus, in Supplemental Figure 14, we propose a working model that integrates these findings.

Donor antigen in conjunction with anti-CD40L costimulation blockade in both murine and nonhuman primate models is an effective tolerizing regimen in a variety of transplant settings (29, 65), whereby anti-CD40L costimulation blockade abrogates the receipt of a necessary stimulatory signal to prevent activation of alloreactive T cells (37). The addition of donor antigen has been shown to promote a stronger and more robust form of tolerance (26, 43), though the reasons for this are still largely hypothetical. Some studies have begun exploring the influence of dead cell clearance on DCs (58, 66), though exploration of this process within specific DC subsets is in its infancy. Importantly, the context of cell engulfment by DCs appears to be critical given that efferocytosis of infected versus sterile apoptotic cells was shown to result in distinct migratory and stimulatory capacities, as indicated by markers of activation and cytokine production (66). Our results reveal that uptake of donor antigen administered during a DST + CoB tolerizing regimen by cDC1s in an efferocytic process may be inducing a regulatory signaling cascade, leading to increased TGF-β1 expression and subsequent induction of antigen-specific CD4^+^CD25^+^FoxP3^+^ T cells. It is well established that CD4^+^CD25^+^Foxp3^+^ Treg cells play a pivotal role in both initiating and sustaining allograft tolerance. The observed reduction of Tregs in cDC1-KO recipients demonstrates that the cDC1 population critically contributes, at least in part, to the phenomenon of allograft tolerance.

Efferocytosis has already been shown to result in increased TGF-β1 production in macrophages (67) in a pathway dependent upon apoptotic cell metabolites (68); thus, it is possible a related mechanism is present within cDC1s. The importance of mitochondria as signaling organelles within the immune system is profound (61), and the shift from glycolytic to mitochondrial metabolism resulting in the promotion of a reparative or antiinflammatory polarization has been reported in a number of immune cells including macrophages (19) and Tregs (64). A similar paradigm has been described in in vitro studies of DC metabolism (69), but importantly, many of these studies rely upon the use of GM-CSF BM-derived DCs, which are functionally distinct from their in vivo or Flt3L-derived cDC counterparts (49, 50). However, our targeted studies of cDC1s genetically deficient in mitochondrial metabolism through deletion of complex III subunits are in concordance with these findings, which may further affirm the conserved nature of these signaling pathways.

Our experimentation focused on the overall metabolic state of in vivo cDC1s after DST + CoB exposure, whereas recent work has described the use of tryptophan metabolism by cDC1s after LPS stimulation to communicate a tolerogenic signal through specific metabolites to cDC2s (17). Although LPS is considered a highly inflammatory stimulus in comparison with that of efferocytosis, it is interesting to contemplate how related metabolite signaling or byproducts of the electron transport chain, such as mitochondrial ROS, may be contributing to this induction of TGF-β1 expression in cDC1s. It will be necessary to define the specific signaling mechanism being utilized from this increase in cDC1 mitochondrial metabolism in order to target this pathway to promote or enhance a desired immunoregulatory or tolerogenic state.

Like most studies, the conclusions of our experiments have limitations. Our early studies utilized a well-accepted murine model for specific deletion of cDC1s, and both the creators of this model (30) and our study observed no statistical differences in other immune cell populations of cDC1-KO and WT mice in the naive state, but it is not impossible to imagine a situation where cDC1 deficiency has an impact on the homeostatic immune system whereby cells are easier to activate after inflammatory insult. However, given our studies in which specific deletion of pathways expressed within cDC1s gave corroborating results, this is of less concern. Additional aspects of our working model warrant future study. For example, the metabolic shift toward oxidative phosphorylation occurred after DST + CoB exposure, which we hypothesize to be the result of cDC1 efferocytosis of donor antigen, but we did not explore which cDC1 apoptotic cell receptors may be necessary for such cellular uptake and downstream signaling. Relatedly, it is very plausible that the electron transport chain and mitochondrial metabolism are critical to other aspects of cDC1 function, such as migration, motility, and antigen presentation, in addition to promoting expression of TGF-β1. It will be necessary to untangle which signaling features of mitochondrial metabolism are necessary for our findings to be applied in additional biological states or utilized in therapeutic interventions. Finally, we observed an increase in intracellular IFN-γ and IL-12 in allograft CD4^+^ T cells by flow cytometry on day 65 after transplantation in cDC1-KO mice compared with B6 controls, implicating these cells as effectors of the earlier rejection of cDC1-KO cardiac allografts (unpublished data). However, the effect of cDC1 loss or their functional disruption and the subsequent impact on effector CD4^+^ and CD8^+^ T cells throughout the life of the transplant requires further investigation.

In summary, our studies identify cDC1s as critical to the induction of antigen-specific tolerance and immune regulation in the setting of alloantigen. Exposure of cDC1s to alloantigen triggers a transcriptional reprogramming through which cDC1s are polarized toward mitochondrial metabolism and subsequent TGF-β1 expression. Loss of either cDC1 TGF-β1 or cDC1 mitochondrial metabolism results in a loss of the ability to induce antigen-specific CD4^+^CD25^+^FoxP3^+^ T cells. Additional studies are underway to further elucidate the basic molecular mechanisms by which mitochondrial metabolism signaling within cDC1s functions to promote the development of immunoregulatory cell populations as well as the potential of therapeutically targeting these pathways in transplantation and other states of inflammation.

## Methods

### Sex as a biological variable.

Our study examined male and female animals, and similar findings are reported for both sexes.

### Animals.

Eight to 12-week-old C57BL/6J (B6), CD90.1^+^ C57BL/6J (CD90.1^+^), BALB/c, CD45.1^+^ BALB/c (CD45.1^+^), Xcr1^Cre/+^, TGF-β1^fl/fl^, Ova, and OTII mice were obtained from The Jackson Laboratory and bred at Northwestern University. *Irf8* +32^–/–^ cDC1-KO mice (cDC1-KO) were provided by Kenneth Murphy (Washington University, St. Louis, Missouri, USA) and generated as previously described (30). QPC^fl/fl^ mice were provided in-house and generated as previously described (64). Risp^fl/fl^ mice were provided by Paul Schumacker (Northwestern University Feinberg School of Medicine) and generated as previously described (70). Mice were housed in specific pathogen–free conditions in temperature- and humidity-controlled environments and kept on a 12-hour light/12-hour dark cycle with access to standard mouse chow and water ad libitum at Northwestern University.

### PKH-67–labeled splenic cell infusion.

Spleens of BALB/c mice were harvested, RBC-lysed, and twice washed with PBS. Cells were counted and labeled with PKH-67 green fluorescence utilizing a general cell membrane labeling kit (MilliporeSigma). Labeled cells were counted and resuspended at a concentration of 5 × 10^7^ cells in 120 μL PBS and i.v. injected. Recipient mice were harvested 18 hours after injection and spleens processed for flow cytometry.

### Murine heart transplant and tolerance induction strategy.

Age- and sex-matched BALB/c mice served as donors to recipient mice, all on a B6 background. Heterotopic cardiac allografts were implanted into mice as previously described (31) in collaboration with the Northwestern Microsurgery Core. Briefly, donor ascending aorta and pulmonary arteries were sutured to recipient abdominal aorta and inferior vena cava to achieve full anastomosis. A hyporesponsive immunological state was induced through administration of anti-CD154 (Bio X Cell; 500 μg per mouse, i.v. on day 0, i.p. on day 7) and donor-specific transfusion (2 × 10^7^ million donor splenocytes, i.v. on day 0) to recipients, similar to previously described procedures (65, 71). Allografts were monitored for signs of rejection by manual palpation every other day. Allograft rejection was defined as complete cessation of heartbeat (32).

### Echocardiography.

Cardiac allograft function was assessed by 2D M-mode echocardiography (Vevo from VisualSonics) as previously described (32, 72) on day 42 after transplantation. M-mode images were collected at the level of the papillary muscles, and measurements were made in 3 consecutive cardiac cycles and averaged for analysis. Left ventricular end-diastolic and end-systolic dimensions were determined from M-mode tracings, and fractional shortening was measured as an indicator of graft function.

### Histology.

Mice were euthanized and perfused with PBS to remove peripheral cells. Allografts were excised and atria removed before embedding tissue in OCT compound in a cryomold over dry ice. Allografts were sectioned serially along the transverse plane from allograft apex to base at a thickness of 10 μm on a Leica cryostat and placed on Superfrost Plus–coated slides (Thermo Fisher Scientific). Before staining, frozen sections were fixed with 4% phosphate-buffered formalin and washed with water. Sections were stained using a routine H&E staining protocol.

### Flow cytometry.

Mice were euthanized and host orthotopic hearts and cardiac allografts were extensively flushed with saline to remove peripheral cells, then excised, minced with fine scissors, and digested with collagenase and DNase at 37°C for 30 minutes as described previously (73). In experiments requiring assessment of DCs, spleens were excised and injected with 3 mL of collagenase D (1 mg/mL) and DNase I (20 μg/mL). After 5 minutes, injected spleens were minced and allowed to incubate at 37°C for 20 minutes followed by addition of 5 mM EDTA during the last 5 minutes (74). All tissues were homogenized by pipetting and passed through a 40 μm cell strainer. Erythrocytes were lysed and total viable cell numbers were determined by Trypan blue staining or Nexcelom Cellometer. Cells were then incubated with Fc Block (BioLegend) for 15 minutes and labeled with fluorescently conjugated antibodies for 30 minutes on ice in the dark. For cells requiring storage or intracellular staining, cells were permeabilized and fixed by suspending in 4% paraformaldehyde for 10 minutes and washed with perm/wash buffer (BD Biosciences). Cells were stained with intracellular antibodies in perm/wash buffer, washed, and resuspended in stain wash buffer for analysis. For assessment of cellular FoxP3, cells were stained with extracellular antibodies as previously described, followed by use of True-Nuclear Transcription Factor buffer set kit (BioLegend) and its associated staining protocol. For assessment of mitochondrial mass, cells were stained with extracellular antibodies as previously described, followed by use of MitoTracker Green FM (Thermo Fisher Scientific) according to the manufacturer’s protocol. Flow cytometry was performed on an LSR Fortessa X-20 instrument (BD Biosciences), and data were analyzed by FlowJo10.8.1 software (Tree Star). Antibodies utilized are listed in the table in the supplemental materials.

### Donor-specific antibody measurement.

Donor-specific antibodies were measured in transplant recipients (all B6 background receiving BALB/c allografts) as described previously (75). Briefly, blood was collected from recipients at predetermined time points, and serum was isolated by centrifugation. Serum samples were incubated with BALB/c splenocytes for 1 hour on ice. Cells were washed and stained with B220 and IgG fluorescent antibodies listed in the table in the supplemental materials. MFI of IgG on live B220-negative cells was measured by flow cytometry. BALB/c cells incubated without serum were utilized as a negative control, and values are reported as a fold-change from the negative control MFI.

### Donor splenocyte and costimulation blockade infusion (DST + CoB).

A hyporesponsive immunological state was induced through administration of anti-CD154 (Bio X Cell; 500 μg per mouse) and donor-specific transfusion (2 × 10^7^ million splenocytes) through i.v. injection to recipients as previously described (65, 71). Splenocytes were obtained from BALB/c, CD45.1^+^ BALB/c, or ovalbumin-expressing mice depending upon the experiment.

### Adoptive transfer of OTII cells with persistent antigen stimulation.

Adoptive transfer and persistent antigen stimulation were performed as previously described (47, 76). Briefly, OTII (CD90.1^+^ TCR Vb5^+^) cells were isolated from the spleen, RBC-lysed, and magnetically enriched for CD4^+^ cells (STEMCELL Technologies). Enriched CD4^+^ cells were counted and 3 × 10^6^ cells resuspended into 120 μL PBS and adoptively transferred into recipient mice by retro-orbital injection on day –1. Recipients then received standard DST + CoB treatment on day 0, which included administration of anti-CD154 (Bio X Cell; 500 μg per mouse) and donor-specific transfusion (2 × 10^7^ million ovalbumin-expressing splenocytes) through i.v. injection. For persistent antigen infusion, RBC-lysed splenocytes from one-quarter to one-sixth of the spleen of ovalbumin-expressing mice were resuspended in 200 μL PBS and i.p. injected every 48 hours after day 0.

### BM-derived DC culture.

BM-derived DCs were generated as previously described (50). BM cells were extracted from femurs and tibias and RBCs lysed. Cells were cultured for 7 days at a plating density of 1.5 × 10^6^ cells in RPMI 1640 medium with 10% FBS containing recombinant mouse 100 ng/mL Flt3L (BioLegend) at 37°C in 5% CO_2_. Half media was exchanged on day 3 with fresh media containing 50 ng/mL of Flt3L. Cells were replated at equivalent densities on day 7 with fresh media containing 50 ng/mL of Flt3L and experimentation initiated on day 8.

### Single-cell sequencing sample preparation and harvest.

Splenic samples for single-cell sequencing were harvested and prepared as previously described (58). Spleens of recipient mice were harvested 48 hours after saline or DST + CoB infusion (3 biological replicates per infusion condition) and subjected to collagenase D (300 U/mL) and DNase I (30 U/mL) digestion in HBSS at room temperature for 20 minutes and 1 mM EDTA for 5 minutes. Spleens were mechanically pressed through a 40-μm filter, pooled by condition, RBC-lysed, and filtered. Total viable cell numbers were determined by trypan blue staining. Cells were resuspended at appropriate concentration and each condition separated into 2 distinct enrichment procedures. Cells were independently subjected to CD45^+^ or pan-DC magnetic enrichment kit protocols (STEMCELL Technologies) according to the manufacturer’s instructions. Enriched cell suspensions were counted and re-pooled by condition at a 1:1 ratio for library preparation.

### Single-cell library preparation and sequencing.

Single-cell mRNA sequencing libraries were prepared utilizing the 10x Genomics Chromium Next GEM single-cell 3′ library and gel bead kit (v3.1) pipeline following the manufacturer’s protocols. A targeted number of 9,000 cells from the DC-enriched cell suspension were loaded per experimental condition and RNA quality confirmed utilizing Northwestern’s NUSeq Core Facility Agilent Bioanalyzer high-sensitivity chip and KAPA library quantification kits for the Illumina platform (KAPA Biosystems). Libraries were sequenced on the HiSeq platform (Illumina) to a read depth of approximately 25,000 reads per cell by Novogene.

### Single-cell sequencing analysis and visualization.

Raw fastq files were analyzed using Cell Ranger version 4.0.0 (10x Genomics) (77), and barcode-gene matrices were analyzed using Seurat R package (v4.3.0) (78). Low-quality cells were removed as identified by a low unique molecular identifier count (<500), low gene number (<250), or a high ratio of mitochondrial reads (>0.2). Cell cycle heterogeneity was evaluated by cell cycle phase scoring and regressed (79). Datasets for control and DST + CoB conditions were integrated using biological state “anchors” to minimize batch affects and allow for comparative analysis across conditions (80). Normalization and principal component analysis were utilized to reduce dimensionality and UMAP to visualize and unbiasedly cluster cells. Cluster identification was performed using an unbiased SingleR algorithm, which compares experimental data to reference transcriptomic datasets (81). SingleR identifications were confirmed through assessment of canonical immune cell markers conserved across conditions.

### Differential gene expression and pathway enrichment analysis.

Differential expression tests to identify differentially expressed genes (DEGs) within clusters between experimental conditions were performed utilizing R packaged DESeq2 (v1.38.2) (82). DEGs with adjusted *P* values less than 0.05 were input into g:Profiler to identify enriched molecular pathways. Gene sets from Gene Ontology biological processes were used (60).

### Isolation and sorting of splenic cDC1 population.

Splenic tissue of WT or DST + CoB–treated mice was harvested and subjected to tissue digestion as previously described in *Flow cytometry*. After RBC lysis and filtration, whole murine spleens were resuspended in 200 μL MACSQuant Tyto Running buffer (Miltenyi Biotec) and cells stained with MHCII, CD11c, XCR1, and CD172 antibodies for 20 minutes on ice in the dark. Additional MACS Quant Tyto Running buffer was added (1 mL total volume) and cells stained with DAPI for 10 minutes. The volume of cell suspension was increased to approximately 6 mL, and cells were subjected to sorting on MACSQuant Tyto Cell Sorter, collecting events that were XCR1^+^CD172^–^ (gating strategy and protocol in Supplemental Figure 8). The positive fraction of cells from the first sort was subsequently collected and resuspended in appropriate volume to achieve a triggered events/second rate of approximately 3,000 for the second “purity” sort. The positive fraction of collected cells after the second sort was assessed for the purity of cDC1s on an LSR Fortessa X-20 instrument (BD Biosciences) and subjected to downstream metabolic analysis.

### Coculture of DCs and CD4^+^T cells.

Naive CD4^+^ T cells were isolated from lymph nodes using the MojoSort naive CD4^+^ T Cell Isolation kit (BioLegend) following the manufacturer’s protocol. Purified naive CD4^+^ T cells were plated 1 × 10^6^ cells/mL in 100 μL in a 96-well plate. CD4^+^ T cell wells were precoated with 2 μg/mL anti-mouse CD3 and CD28 antibodies in PBS, incubated overnight at 4°C, and washed twice to remove residual antibody. Flt3L BMDCs were generated as previously described in *BM-derived DC culture* and were stimulated with or without saline or UV-irradiated allogenic DST from a CD45.1 BALB/c mouse at a 2:1 ratio for 48 hours. BMDCs were added to the culture to coculture with naive CD4^+^ T cells for a final ratio of 1:4 in 200 μL. Next, 5 ng/mL of recombinant Il-2 (BioLegend) was used to supplement the cultures. Naive CD4^+^ T cells were also cultured independently; without DCs, anti-CD3/CD28 only; or with a combination of anti-CD3/CD8, rIl-2, and 5 ng/mL TGF-β (BioLegend) to induce activation (CD25^+^FoxP3^–^) or Treg cell (CD25^+^FoxP3^+^) phenotypes as positive and negative controls for flow cytometric gating. Either saline or 25 μg/mL of anti-mouse TGF-β 1,2,3 neutralizing antibody (Invitrogen) was added to DST-treated BMDC cocultures. Cocultures were maintained for 5 days in RPMI 1640 medium with 10% FBS, and cells were harvested for multicolor flow cytometry as previously described in *Flow cytometry*.

### Oxygen consumption rate and metabolic measurements.

Sorted splenic cDC1s from mice treated with PBS or DST + CoB were plated on Seahorse XF HS Culture Miniplates coated with CellTak. Experiments were conducted in phenol-free RPMI 1640 XF assay medium containing 11 mM glucose, 2 mM glutamine, 1 mM pyruvate, 2% FBS, and 1 mM HEPES buffered to a pH of 7.4 and analyzed using a Seahorse XF HS Mini Analyzer (Agilent Technologies). Where indicated, the following were injected: BAM 15 (2 μM) to uncouple ATP synthesis, antimycin A (1 μM) to block mitochondrial complex III, piericidin A (1 μM) to block mitochondrial complex I, and 2-deoxy-D-glucose (25 mM). Basal OCR, max respiration, and respiratory capacity were generated by Seahorse XF HS Mini Analyzer and analyzed in GraphPad Prism 9.

### Statistics.

Analysis was performed with GraphPad Prism 9 and 10. Allograft survival was calculated by log-rank analysis. Comparisons between 2 groups were done using a 2-tailed unpaired *t* test with a 95% CI. For comparisons of more than 2 variables, 1-way ANOVA was used with a 95% CI, and Tukey’s test was used to correct for multiple comparisons. The experimental sample size is indicated in each figure and represents pooled data from 2 or more independent experiments. Data are presented as the mean ± SD. The criteria for significant differences are indicated in figure legends. Statistical significance for all figures was set at *P* less than 0.05.

### Study approval.

All animal studies were conducted in accordance with guidelines and protocols approved by the IACUC of Northwestern University.

### Data availability.

Values for all graph data points are reported in the Supporting Data Values file. The datasets generated and analyzed for this study have been uploaded into NCBI’s Gene Expression Omnibus (GEO). The control infusion dataset can be found under GSE223921 as it was published and analyzed as a part of an earlier study in the laboratory (58). The DST + CoB dataset can be found under GSE251761.

## Author contributions

SLS, RTLJ, SEW, MLA, HI, JTD, NSC, and EBT designed the experimental methods. SLS, LZ, RTLJ, KG, NLM, and SEW conducted experiments. SLS performed bioinformatic analysis of sequencing data. SLS, LZ, RTLJ, and EBT cowrote the manuscript text. SLS, LZ, and RTLJ prepared figures. All authors reviewed the manuscript.

## Supplementary Material

Supplemental data

Supporting data values

## Version 1 07/11/2025

In-Press Preview

## Version 2 09/16/2025

Electronic publication
