# Supplementary material for: Treg activation during allograft tolerance induction requires mitochondrion-induced TGF-β1 in type 1 conventional dendritic cells
Source: J Clin Invest. 2025 Jul 10;135(18):e178960. doi: 10.1172/JCI178960 (PMC12435855; doi:10.1172/JCI178960)
Supplement: Supplemental data [file jci-135-178960-s070.pdf]

SUPPLEMENTAL FIGURES BELOW

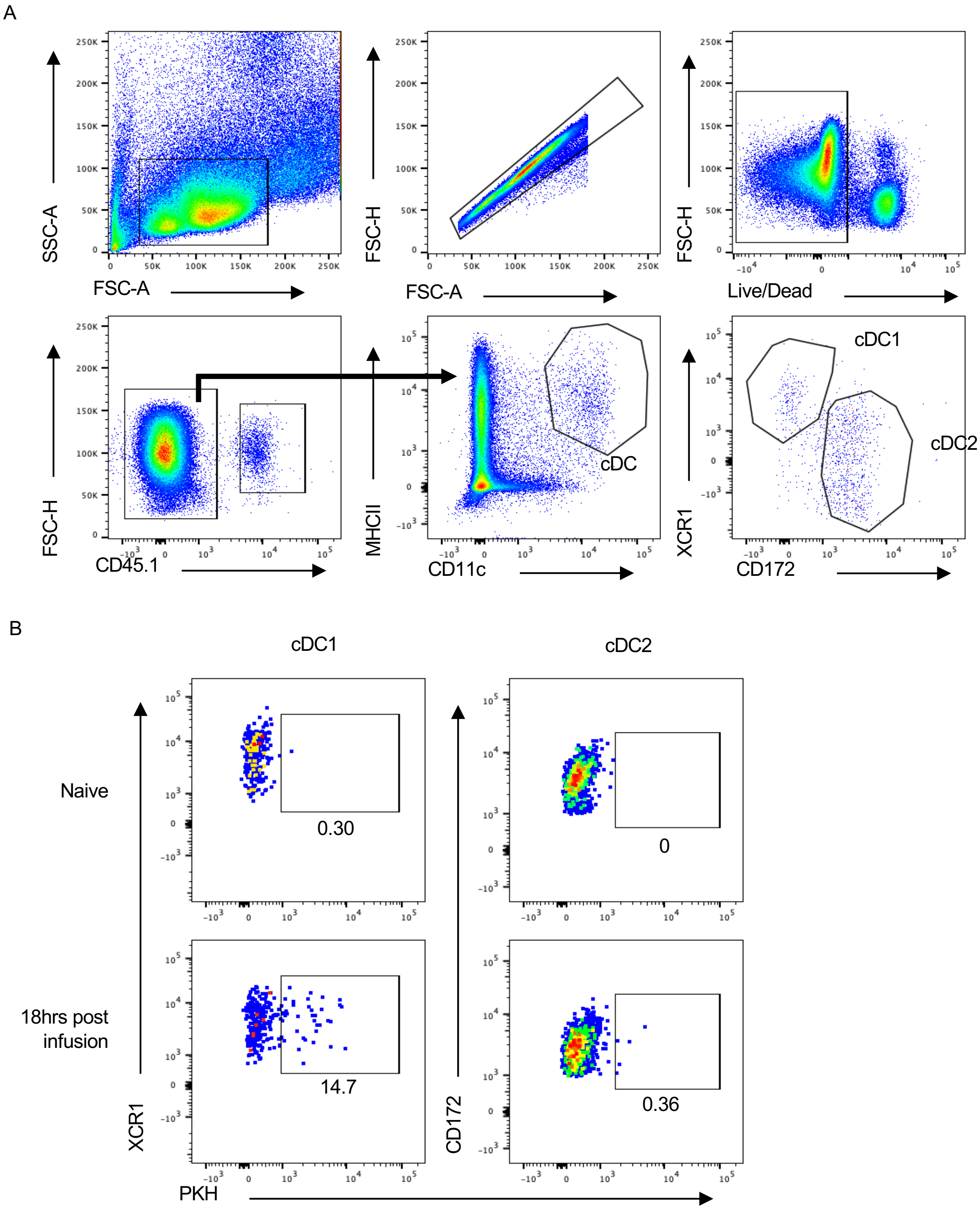

Supplemental Figure 1

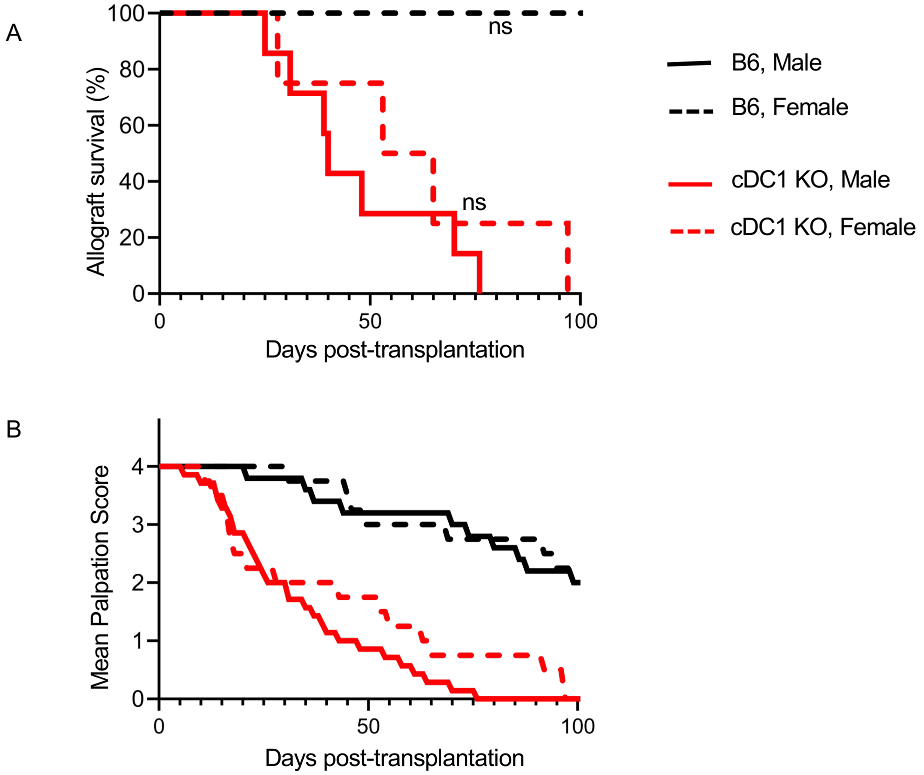

Supplemental Figure 2

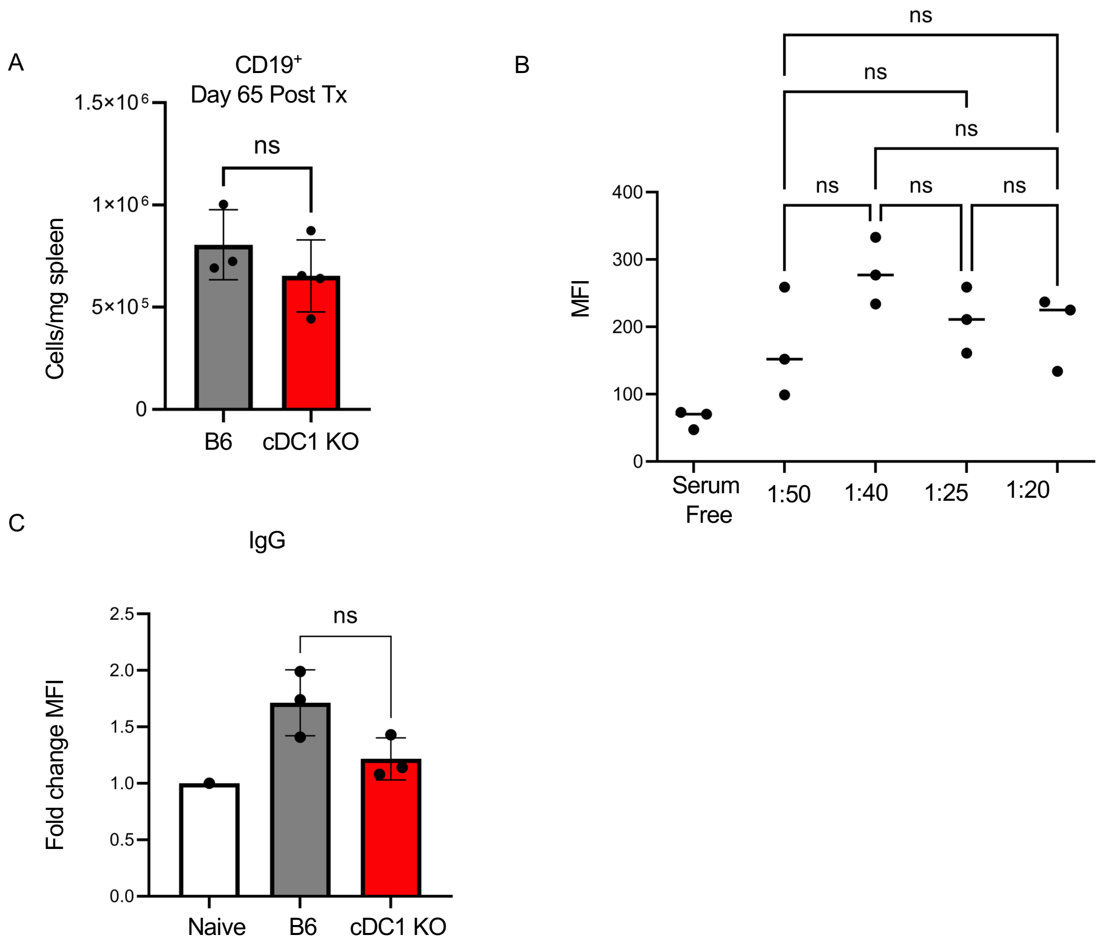

Supplemental Figure 3

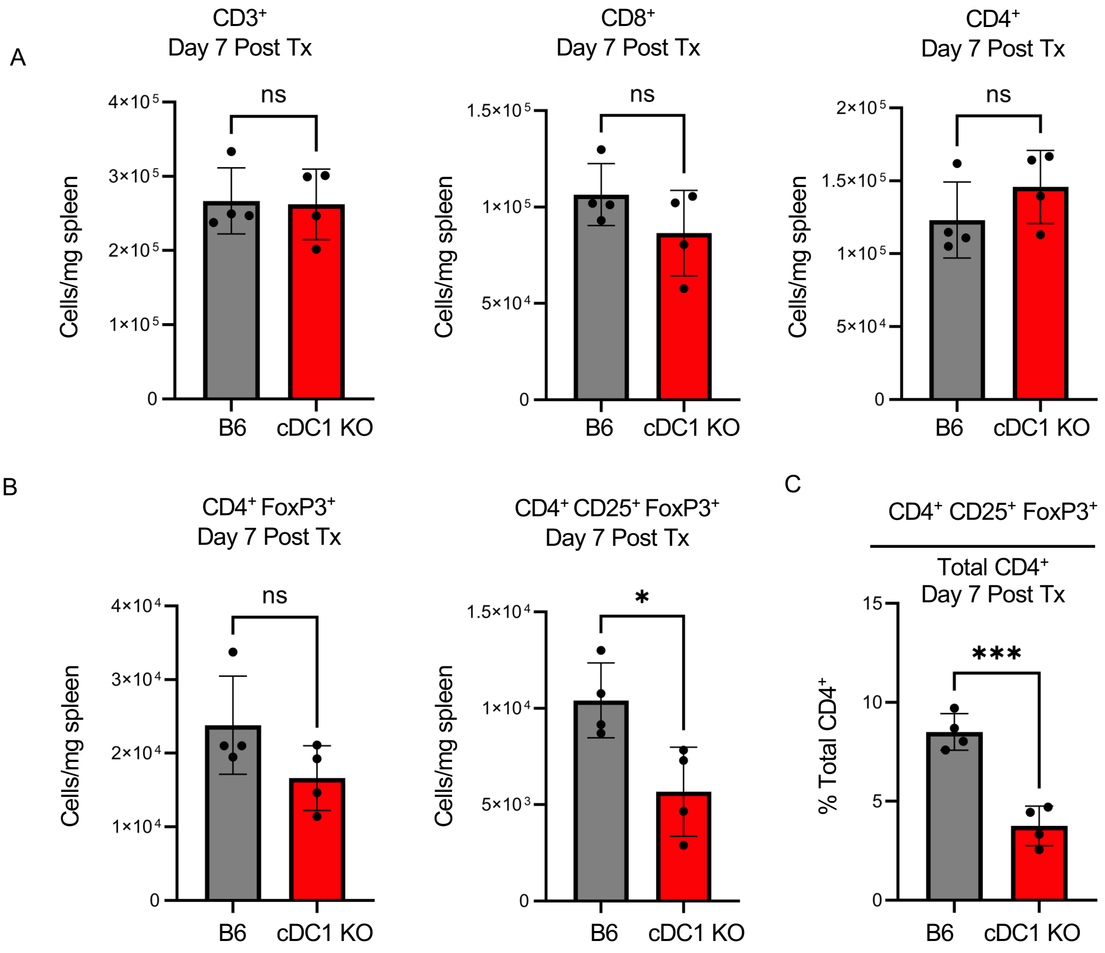

Supplemental Figure 4

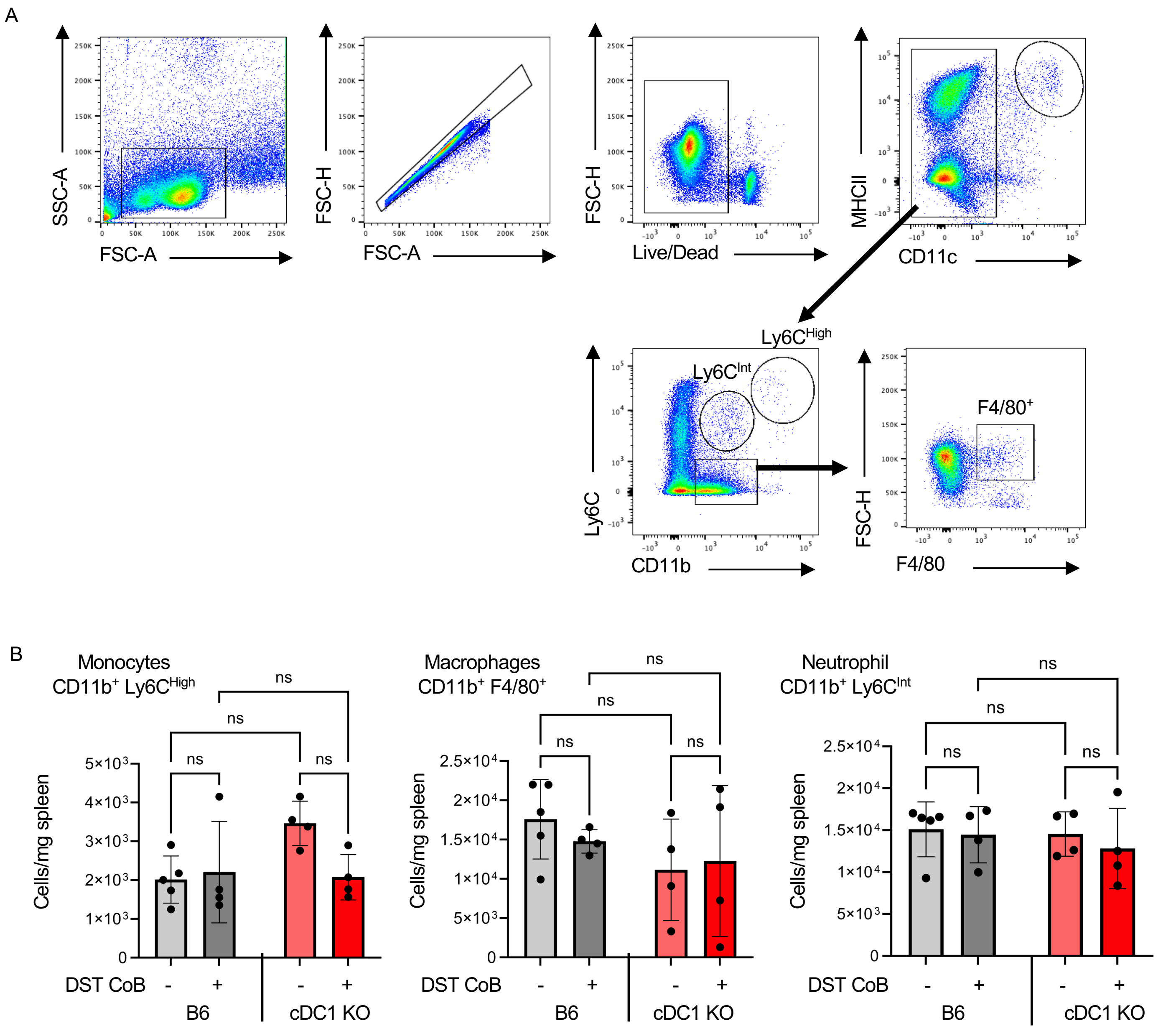

Supplemental Figure 5

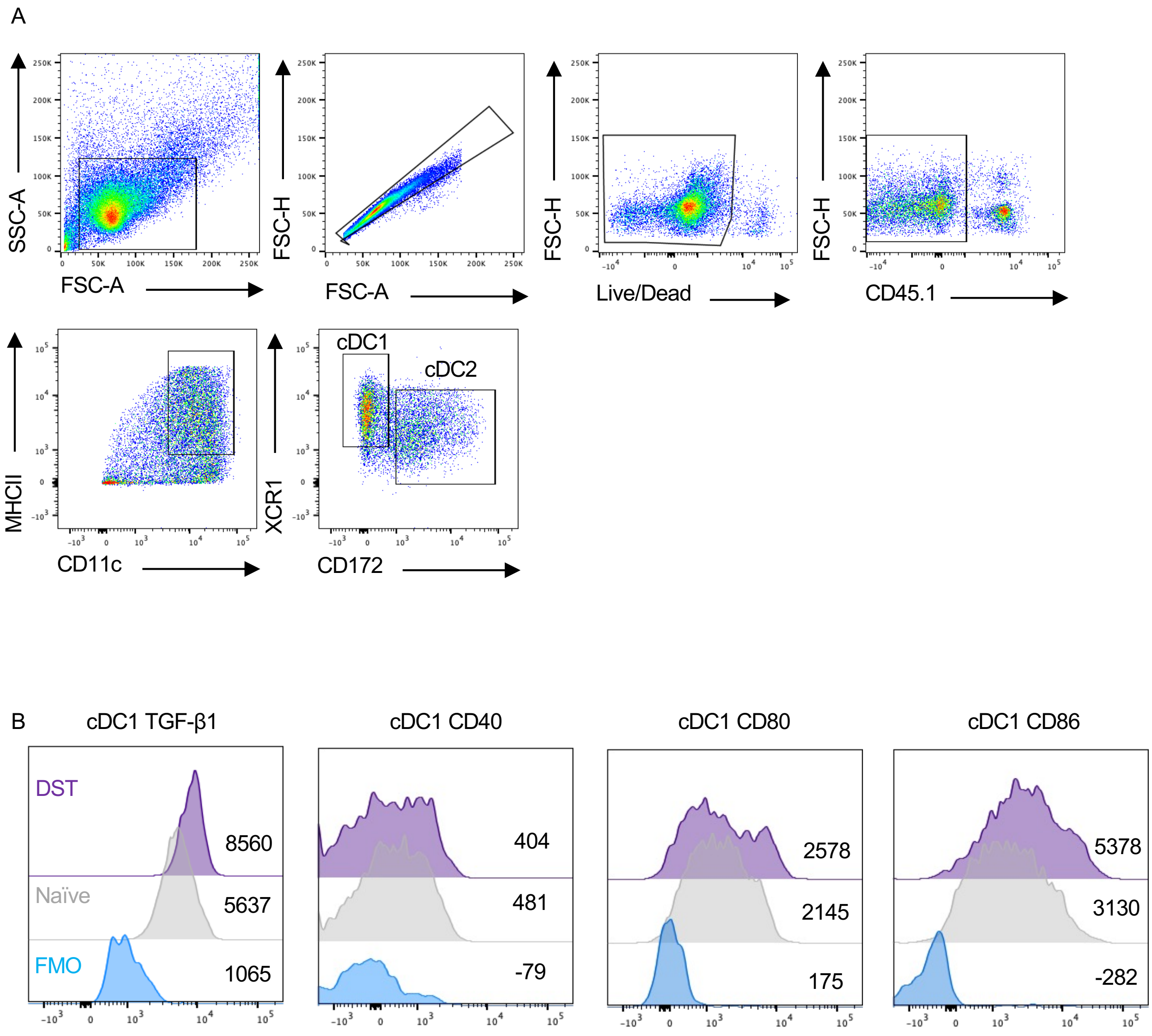

Supplemental Figure 6

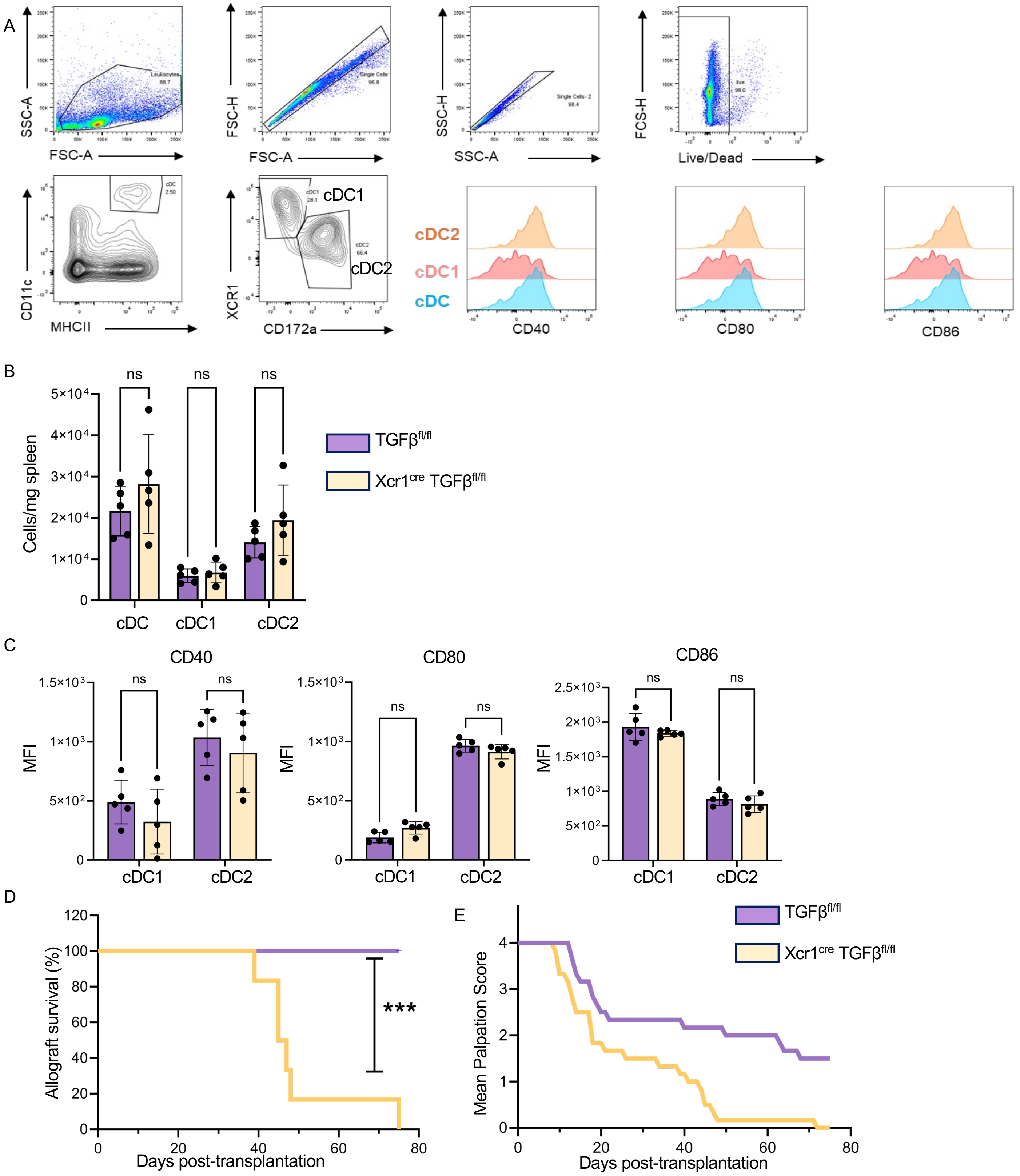

Supplemental Figure 7

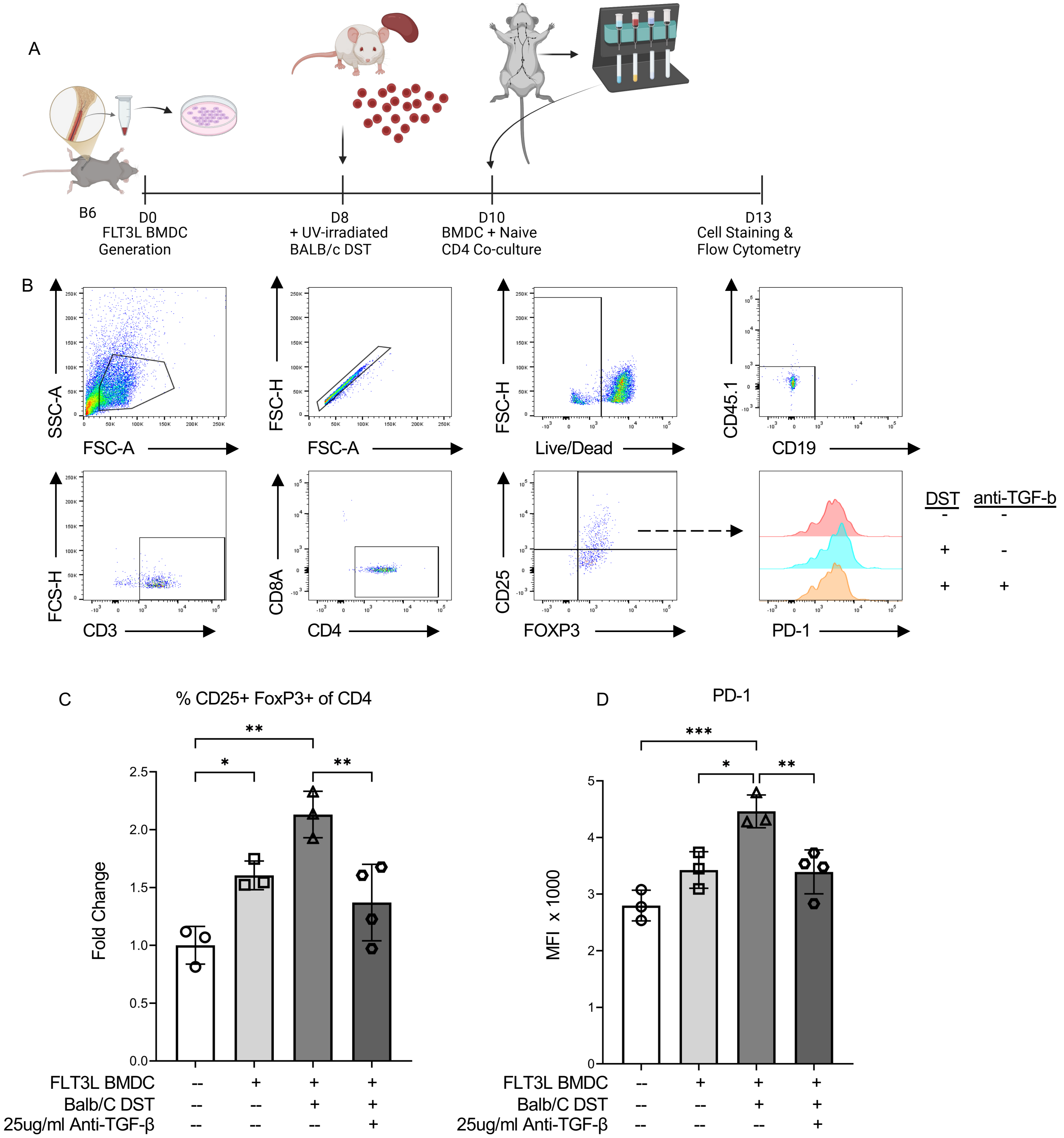

Supplemental Figure 8

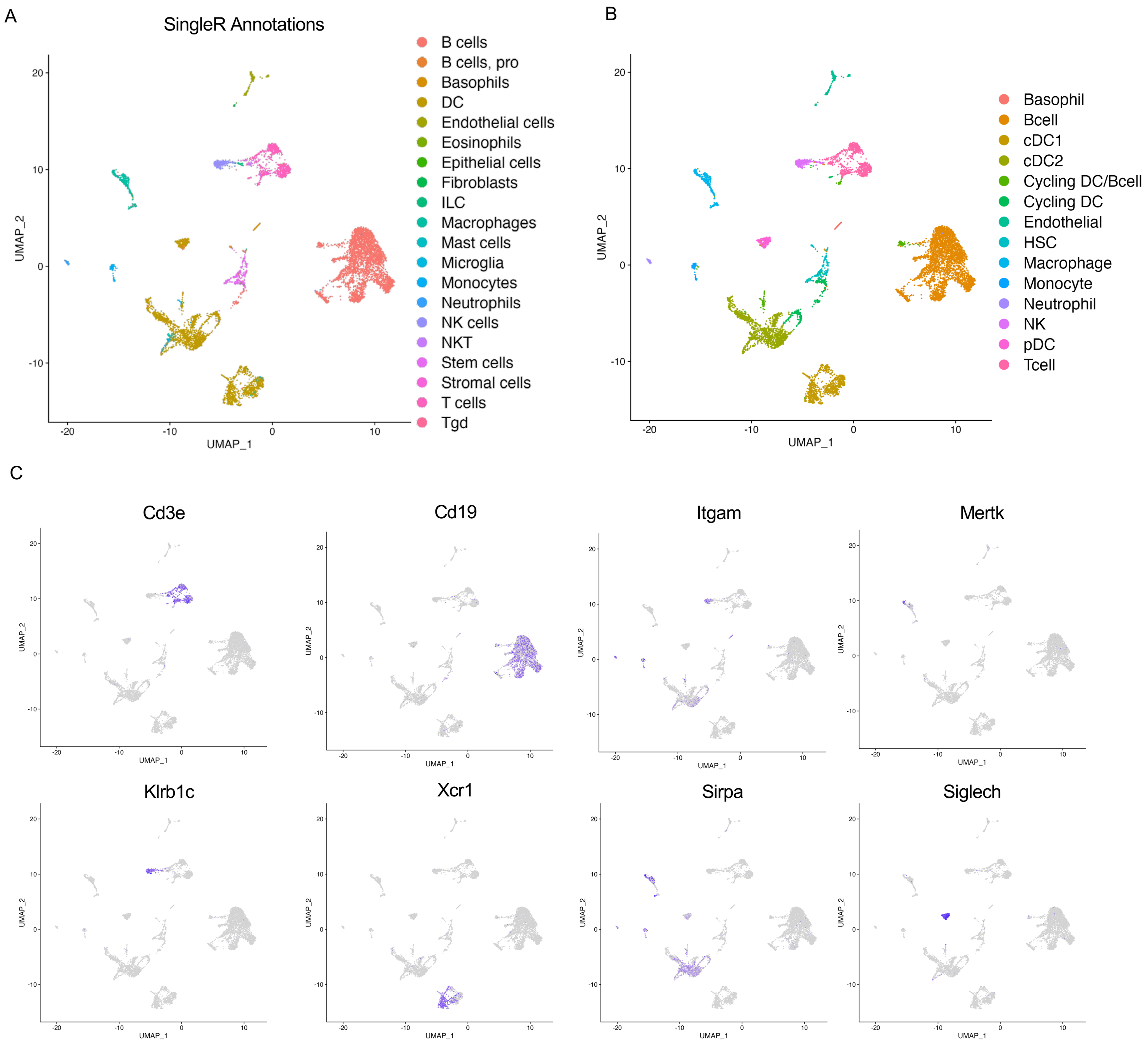

Supplemental Figure 9

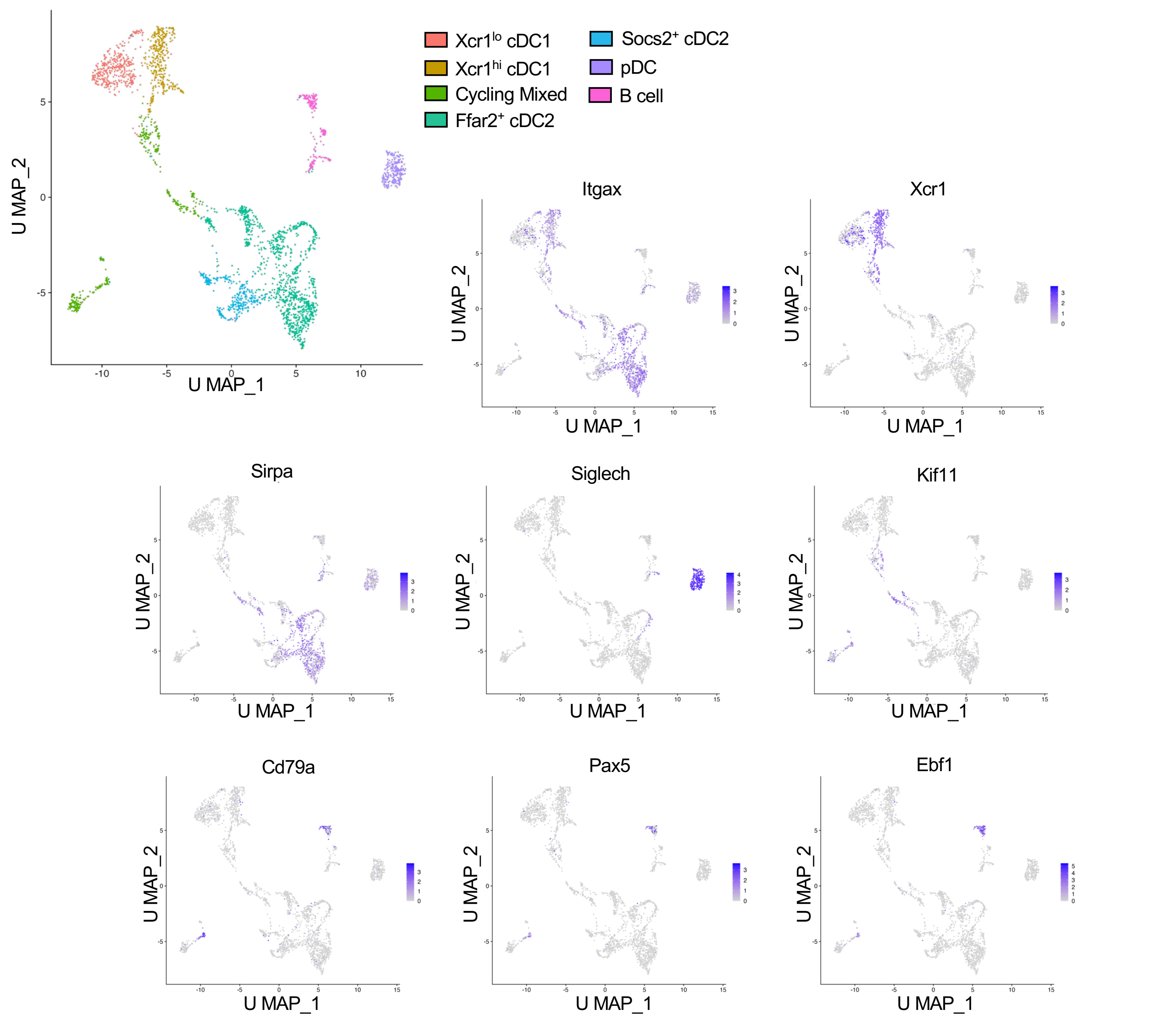

Supplemental Figure 10

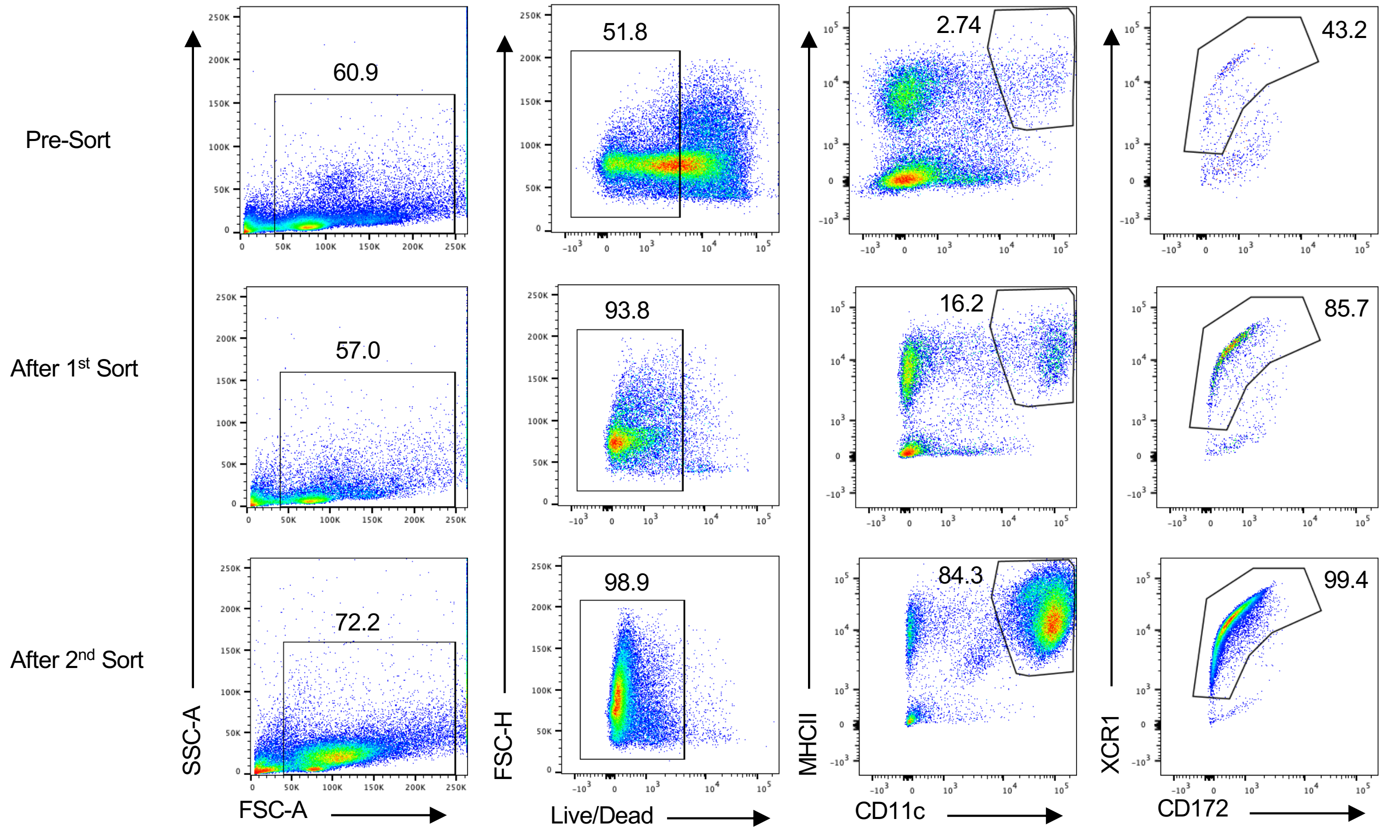

Supplemental Figure 11

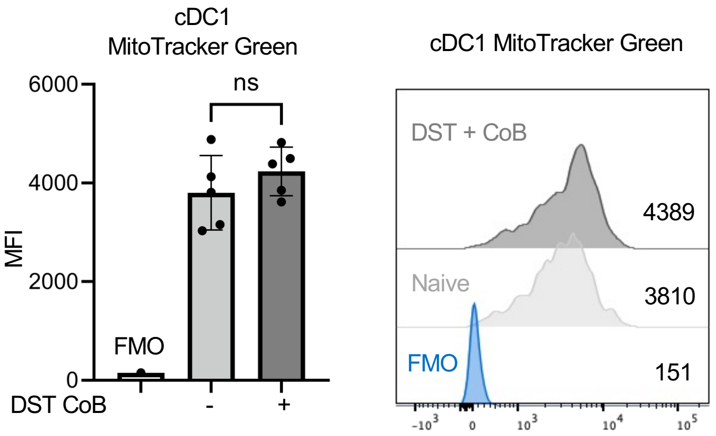

Supplemental Figure 12

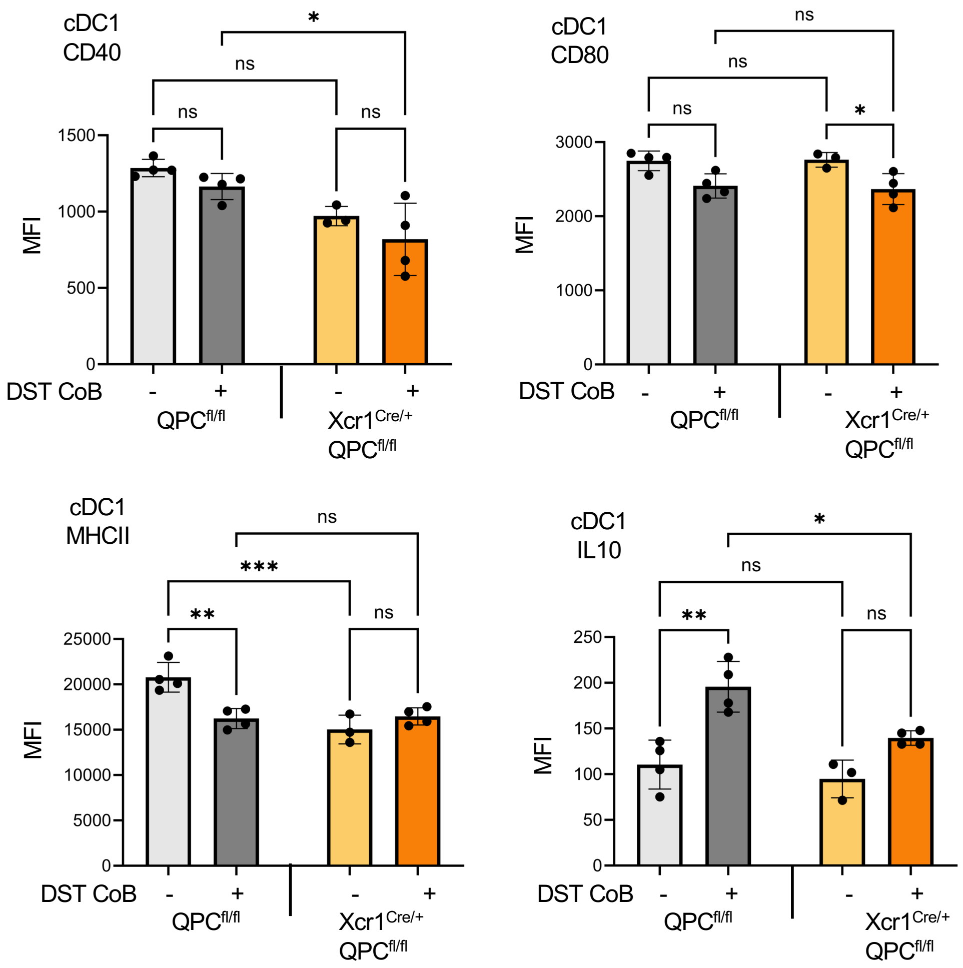

Supplemental Figure 13

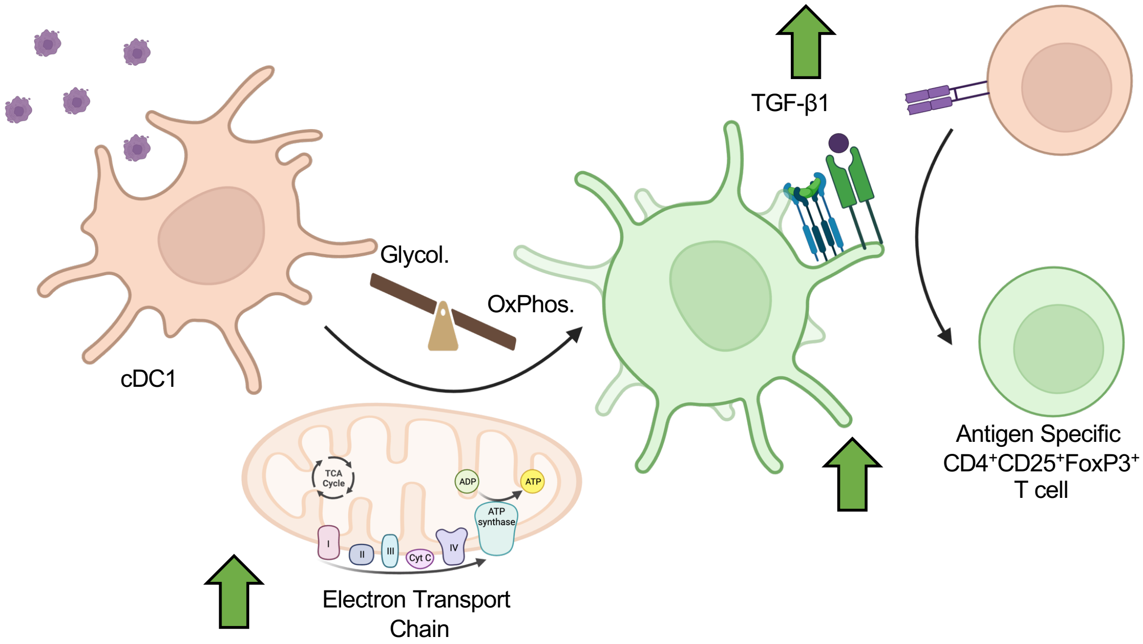

Supplemental Figure 14

Supplemental Figure Legends

Supplemental Figure 1. Localization of membrane labeled alloantigen to cDC1s. (A) Flow cytometry gating strategy to identify cDC1 and cDC2 cells in murine spleens. **(B)** $CD45.1^+$ BALB/c splenocytes were membrane labeled with PKH67 and injected into B6 mice and spleens harvested 18 hours later. Representative flow cytometry plots of recipient $CD45.1^-$ cDC1 and cDC2 cell populations in naïve and membrane labeled alloantigen injected mice.

Supplemental Figure 2. Cardiac allograft survival and mean palpation scores by sex. (A) Survival of BALB/c cardiac allografts in B6 and cDC1 KO mice as determined by manual palpation with rejection occurring at complete cessation of heartbeat and a palpation score of 0, separated by sex. n = 4-7 per group. Ns, no significance by log-rank test. **(B)** Mean palpation score of BALB/c cardiac allografts in B6 and cDC1 KO mice, separated by sex. n = 4-7 per group.

Supplemental Figure 3. Contribution of B cells and donor specific antibodies to cardiac allograft rejection of cDC1 deficient mice. (A) Quantification of $CD19^+$ B cells in spleens of cDC1 KO and B6 mice 65 days after cardiac transplantation. n = 4 per group. Ns, no significance by 2-tailed unpaired t test. (**B**) Median fluorescence intensity of anti-BALB/c IgG in B220 negative splenocytes of BALB/c mice incubated with various dilutions of serum from B6 heart transplant mice. n = 3 samples. Data were shown as mean ± SD. ns, no significant by ANOVA followed by Tukey's test (**C**) Presence of anti-BALB/c antibodies assessed by flow cytometry utilizing serum (1:25 dilution) from transplant recipients at day 65 post transplantation incubated with BALB/c splenocytes. Briefly, after incubation the cells were stained for B220 and IgG. Median fluorescence intensity on B220-negative cells was measured and represented as a fold change from BALB/c splenocytes incubated without serum. n = 3 per group. ns, no significance by 2-tailed unpaired t test.

Supplemental Figure 4. Splenic T cell compartment 7 days after cardiac transplantation. (A) Quantification of $CD3^+$, $CD8^+$, and $CD4^+$ T cells in spleens of cDC1 KO and B6 mice 7 days after cardiac transplantation. n = 4 per group. ns, no significance by 2-tailed unpaired t test. **(B)** Quantification of absolute number of splenic $CD4^+FoxP3^+$ and $CD4^+CD25^+FoxP3^+$ cells 7 days after cardiac transplantation. n = 4 per group. ns, no significance by 2-tailed unpaired t test. **(C)** Proportion of $CD4^+CD25^+FoxP3^+$ cells within total $CD4^+$ splenic cell population. n = 4 per group. ***P < 0.001 by 2-tailed unpaired t test .

Supplemental Figure 5. Splenic innate immune cells in the setting of persistent alloantigen and costimulation blockade. (A) Flow cytometry gating scheme to identify splenic innate immune cells. **(B)** Quantification of absolute number of splenic monocytes ($CD11b^+Ly6C^{High}$), macrophages ($CD11b^+F4/80^+$) and neutrophils ($CD11b^+Ly6C^{Int}$) in naïve and DST + CoB and persistent antigen treated cDC1KO and B6 mice. ns, no significance by ANOVA followed by Tukey's test.

Supplemental Figure 6. Flt3L bone marrow derived DC express conical markers and increase TGF-β1 expression after alloantigen exposure (A) Flow cytometry gating scheme to identify in vitro Ftl3L derived cDC1 and cDC2 cells. **(B)** Representative histogram of *in vitro* cDC1 TGF-β1, CD40, CD80, and CD86 MFI. MFI, mean fluorescence intensity.

Supplemental Figure 7. TGF-β1 deficiency in cDC1 cells did not impact cDC1 development but promote allograft rejection. (A) Flow cytometric gating strategy for identification of cDC1 and cDC2s and MFI (mean fluorescent intensity) of populations. **(B)** Quantification of cDC1 and cDC2 cell populations in spleens of $Xcr1^{cre/+}$ TGF**β**1 $^{fl/fl}$ and TGF**β**1 $^{fl/fl}$ mice. n = 5 per group. ns,

no significance by ANOVA followed by Tukey’s test. **(C)** Expression of CD40, CD80, and CD86 on cDC1 and cDC2 in spleens of Xcr1$^{cre/+}$ TGFb1 $^{fl/fl}$ and TGFb1 $^{fl/fl}$ mice. n = 5 per group. ns, no significance by ANOVA followed by Tukey’s test. FMO, fluorescence minus one. **(D)** Survival of BALB/c cardiac allografts in TGFβ1$^{fl/fl}$ and Xcr1$^{cre/+}$ TGFβ1 $^{fl/fl}$ mice as determined by manual palpation with rejection occurring at complete cessation of heartbeat and a palpation score of 0. n = 6 per group. ***P < 0.001 by log-rank test. **(E)** Mean palpation score of BALB/c cardiac allografts in TGFβ1 $^{fl/fl}$ and Xcr1$^{cre/+}$ TGFβ1$^{fl/fl}$ mice.

Supplemental Figure 8. Addition of anti-TGF-beta neutralizing antibody ablates DST stimulated DC co-culture induction of CD25+FoxP3+ T cells from naïve CD4 in vitro. (A) Treg induction by DC co-culture. B6 Mouse bone marrow was isolated on D0 and cultured with Flt3L for 8 days. On Day 8 CD45.1+ BALB/c splenocytes were isolated and UV-irradiated and plated with DCs 2:1 for 48 hours to Day 10. On Day 10, naïve CD4 T cells were isolated from B6 lymph node using magnetic sorting and co-cultured for 3 days with plate bound anti-CD3/CD28, IL-2 and in the presence of, saline, unstimulated BMDC, or DST stimulated BMDC or DST stimulated BMDC with anti-TGF-β neutralizing antibody. **(B)** Flow cytometry gating scheme to identify in vitro induced CD25+ FoxP3+ CD4+ T cells. **(C)** Percent CD25+ FoxP3+ of CD4 T cells co-cultured with BMDC stimulated with saline, BALB/c DST, or BALB/c DST with anti-TGF-β presented as Fold Change relative to naïve CD4 stimulated with anti-CD3/28 and IL-2 alone. n= 3-4 per group. *P < 0.05, **P < 0.01, ***P < 0.001 by ANOVA followed by Tukey’s test. **(D)** Expression of PD-1 (CD279) by CD25+FoxP3+ T cells from anti-CD3/CD28 and IL-2 only or with Flt3L BMDC stimulated with saline, BALB/c DST, or BALB/c DST with anti-TGF-β. n= 3-4 per group. *P < 0.05, **P < 0.01, ***P < 0.001 by ANOVA followed by Tukey’s test.

Supplemental Figure 9. Identification of immune cell clusters after single cell sequencing **(A)** UMAP projections and identification of all immune cell clusters by unbiased SingleR algorithm and Immgen reference dataset, color-coded by cluster from single cell experiment whereby B6 mice were treated with BALB/c DST + CoB infusion D0 (IV) or saline control before collection of spleens after 48 hours, enrichment for DCs, and sequencing. **(B)** UMAP projections of final identification of all immune cell clusters, color-coded by cluster. Clusters containing DCs were utilized in downstream analysis. **(C)** Feature plots of canonical immune cell markers to confirm cluster identity of SingleR named clusters.

Supplemental Figure 10. Identification of DC subset cell clusters following single cell sequencing.
(A) UMAP projection and feature plots of gene expression in DC identified and subset clusters for downstream analysis in single cell experiment whereby B6 mice were treated with CD45.1+ BALB/c DST + CoB infusion D0 (IV) or saline control before collection of spleens after 48 hours, enrichment for DCs, and sequencing. Canonical markers for DCs (*Itgax*), cDC1s (*Xcr1*), cDC2s (*Sirpa*), pDCs (*Siglech*), cell cycling (*Kif11*), and B cells (*Cd79a, Pax5, Ebf1*) were used to confirm cluster identity.

Supplemental Figure 11. Pure cDC1 population can be obtained from splenic tissue using low pressure sorter. Following splenic tissue digestion and labeling with Live/Dead, CD11c, Xcr1, and CD172 antibody a very small proportion of cDC1s (Xcr1^{+}CD172^{-}) are present at pre-sort. Following a first “bulk” sort, there is an increased proportion of cDC1 cells after which the positive cell fraction is subjected to a second “purity” sort. After the second sort, there is a highly pure (>90%) population of live cDC1s isolated from splenic tissue that can be subjected to downstream analysis.

Supplemental Figure 12. Mitochondrial mass of splenic cDC1s is not affected by exposure to alloantigen in the setting of costimulation blockade *in vivo*. B6 mice were treated with DST + CoB and spleens harvested 48 hours after infusion. Cells were stained with MitoTracker Green to obtain a measure of mitochondrial mass in addition to flow cytometry antibodies that would allow for identification of the cDC1 population. MFI of MitoTracker Green was assessed within cDC1s ($MHCII^{+}CD11c^{+}Xcr1^{+}CD172^{-}$). Representative histogram of cDC1 MitoTracker MFI is shown. MFI, mean fluorescence intensity. Data shown as n = 4 per group. Ns, no significance by 2-tailed unpaired t test.

Supplemental Figure 13. Expression of CD40, CD80, MHCII and IL10 on cDC1s in spleens of $Xcr1^{cre/+}$ QPC $^{fl/fl}$ and QPC $^{fl/fl}$ mice following allogenic cell exposure. n = 3-4 per group. *P < 0.05, **P < 0.01 by 2-tailed unpaired t test. FMO, fluorescence minus one.

Supplemental Figure 14. Working model based on experimental findings. The schematic describes a scenario wherein cDC1 uptake of alloantigen leads to a metabolic shift towards oxidative phosphorylation and an increase in cDC1 TGF-β1 expression during antigen presentation which results in the antigen specific induction of $CD4^{+}CD25^{+}FoxP3^{+}$ T cells.

Flow Cytometry			
Antibody Target	Fluorophore	Clone	Supplier
B220	PE	1D3	BD Horizon
CD64	BV711	X54-5/7.1	BioLegend
CD11b	BV605	M1/70	BioLegend
CD11c	PE-CF594	N418	BioLegend
CD172a	PE-Cy7	P84	BioLegend
CD172a	APC-Cy7	P84	BioLegend
CD19	APC	eBio1D3	BioLegend
CD19	PerCP Cy5.5	6D5	BioLegend
CD19	PE	6D5	BioLegend
CD25	E450	PC61.5	Invitrogen
CD25	BV605	PC61	BioLegend
CD25	PE	PC61.5	eBioscience
CD3	BV711	145-2C11	BioLegend
CD3	BV421	17A2	BioLegend
CD4	PE	GK1.5	BD Pharmingen
CD4	APC Cy7	RM4-5	BioLegend
CD4	APC	GK1.5	Invitrogen
CD4	APC	GK1.5	eBioscience
CD40	PerCP Cy5.5	3/23	BioLegend
CD45.1	PE	A20	BioLegend
CD45.1	FITC	A20	BioLegend
CD45.1	PerCP Cy5.5	A20	BioLegend
CD8a	PerCP	53-6.7	BioLegend
CD8a	PE-CF594	53-6.7	BioLegend
CD8a	PerCP Cy5.5	53-6.7	BioLegend
CD80	BV711	16-10A1	BioLegend
CD86	APC	GL1	BioLegend
CD86	PE	GL1	eBioscience
CD90.1	BV421	OX-7	BioLegend
CD152	Pe Cy7	UC10-4B9	BioLegend
CD154	PeCy7	MR1	BioLegend
F4/80	APC	BM8	BioLegend
FoxP3	FITC	FJK-16s	Invitrogen
FoxP3	AF488	MF-14	BioLegend
IgG1	FITC	RMG1-1	BioLegend
IgG2b	FITC	RMG2b-1	BioLegend
IL-10	BV421	JES5-16E3	BioLegend
Ly6C	PE-Cy7	HK1.4	BioLegend
PD-1	APC Cy7	29F.1A12	BioLegend
PD-1	BV510	29F.1A12	BioLegend
MHCII I-A/I-E	BV421	M5/114.15.2	BioLegend
MHCII I-A/I-E	FITC	M5/114.15.2	BioLegend
TCR Vβ5.1, 5.2	PE	MR9-4	BioLegend
TGF-β1	FITC	TW7-16B4	BioLegend
XCR1	BV650	ZET	BioLegend
XCR1	APC	ZET	BioLegend
Zombie Aqua Fixable Viability Kit	BV510	Cat#: 423102	BioLegend

Chemicals/Recombinant Proteins/Commercial Kits		
MitoTracker Green FM	Cat#: M7514	Invitrogen
PKH67 Green Fluorescent Cell Linker Kit	Cat#: PKH57GL-1KT	Sigma-Aldrich
Chromium Next GEM Single Cell 3' GEM, Library & Gel Bead Kit v3.1	Cat#: 1000128	10x Genomics
Chromium Next GEM Chip G Single Cell Kit	Cat#: 1000127	10x Genomics
Single Index Kit T Set A	Cat#: 1000213	10x Genomics
CellTak	Cat#: 354240	Corning
BAM15	Cat#: SML1760	Sigma-Aldrich
Antimycin A	Cat#: A8675	Sigma-Aldrich
Piericidin A	Cat#: 15379	Cayman Chemical
2-Deoxy-D-glucose	Cat#: D8375	Sigma-Aldrich
RBC Lysis Buffer (10x)	Cat#: 420302	BioLegend
True Nuclear Transcription Factor Buffer Set	Cat#: 424401	BioLegend
Anti-CD154 (Clone: MR-1)	Cat#: BE0017-1	BioXCell
Collagenase D	Cat#: COLLD-RO	Sigma-Aldrich
DNase I	Cat#: 11284932001	Sigma-Aldrich
EasySep Mouse CD4$^+$ Cell Isolation Kit	Cat#: 19852	STEMCELL Technologies
EasySep Mouse Pan-DC Enrichment Kit II	Cat#: 19863	STEMCELL Technologies
Recombinant Mouse Flt3L	Cat#: 550706	BioLegend
MACSQuant Tyto Cartridges HS	Cat#: 130-121-549	Miltenyi Biotec
MojoSort™ Mouse CD4 Naïve T Cell Isolation Kit	Cat#480040	BioLegend
Ultra-LEAF Purified anti-mouse CD3ε Antibody	Cat#100339	BioLegend
Ultra-LEAF Purified anti-mouse CD28 Antibody	Cat#102115	BioLegend
Recombinant Mouse Il-2 (Carrier Free)	Cat #575402	BioLegend
Recombinant Mouse TGF-β1 (Carrier Free)	Cat#763102	BioLegend
TGF-beta1,2,3 Monoclonal Antibody, Functional Grade	Cat#16-9243-85	Invitrogen
